# Supplementary material for: Interplay between mobility, multi-seeding and lockdowns shapes COVID-19 local impact
Source: PLoS Comput Biol. 2021 Oct 14;17(10):e1009326. doi: 10.1371/journal.pcbi.1009326 (PMC8516261; doi:10.1371/journal.pcbi.1009326)
Supplement: S1 Text — (PDF) [file pcbi.1009326.s001.pdf]

# Supporting Information
## *Interplay between mobility, multi-seeding and lockdowns shapes COVID-19 local impact*

M. Mazzoli, E. Pepe, D. Mateo, C. Cattuto, L. Gauvin, P. Bajardi, M. Tizzoni, A. Hernando, S. Meloni, J. J. Ramasco

| Country | Incidence onset | Mortality onset |
|---|---|---|
| England | $10^{-5}$ | — |
| France | $7 \times 10^{-5}$ | $10^{-6}$ |
| Germany | $10^{-5}$ | $2 \times 10^{-6}$ |
| Italy | $2 \times 10^{-5}$ | — |
| Spain | $7 \times 10^{-5}$ | $10^{-6}$ |

Table A: **Table of incidence and mortality thresholds used to determine cases and deaths onsets for each administrative area in each country.**

| Province | Incidence onset | Incidence peak time |
|---|---|---|
| East Anglia | 2020-03-26 | 2020-04-11 |
| Bedfordshire and Hertfordshire | 2020-03-23 | 2020-04-13 |
| Essex | 2020-03-24 | 2020-04-08 |
| West Midlands | 2020-03-16 | 2020-03-31 |
| Shropshire and Staffordshire | 2020-03-22 | 2020-04-22 |
| Tees Valley and Durham | 2020-03-24 | 2020-04-08 |
| Northumberland and Tyne and Wear | 2020-03-21 | 2020-04-08 |
| Cornwall and Isles of Scilly | 2020-03-26 | 2020-04-02 |
| Dorset and Somerset | 2020-04-04 | 2020-04-15 |
| Devon | 2020-04-03 | 2020-04-20 |
| West Yorkshire | 2020-03-23 | 2020-04-07 |
| South Yorkshire | 2020-03-17 | 2020-03-31 |
| North Yorkshire | 2020-03-25 | 2020-05-04 |
| East Yorkshire and Northern Lincolnshire | 2020-03-28 | 2020-04-25 |
| Inner London - West | 2020-03-12 | 2020-04-08 |
| Outer London - West and North West | 2020-03-13 | 2020-04-04 |
| Outer London - South | 2020-03-13 | 2020-04-01 |
| Outer London - East and North East | 2020-03-18 | 2020-04-09 |
| Inner London - East | 2020-03-13 | 2020-04-06 |
| Derbyshire and Nottinghamshire | 2020-03-22 | 2020-04-11 |
| Kent | 2020-03-22 | 2020-04-19 |
| Hampshire and Isle of Wight | 2020-03-21 | 2020-04-10 |
| Lancashire | 2020-03-23 | 2020-04-16 |
| Lincolnshire | 2020-03-30 | 2020-04-18 |
| Cheshire | 2020-03-23 | 2020-04-08 |
| Merseyside | 2020-03-20 | 2020-04-08 |
| Cumbria | 2020-03-20 | 2020-04-12 |
| Greater Manchester | 2020-03-18 | 2020-04-08 |

Table B: **Table of epidemic features for NUTS2 of England, when available.**

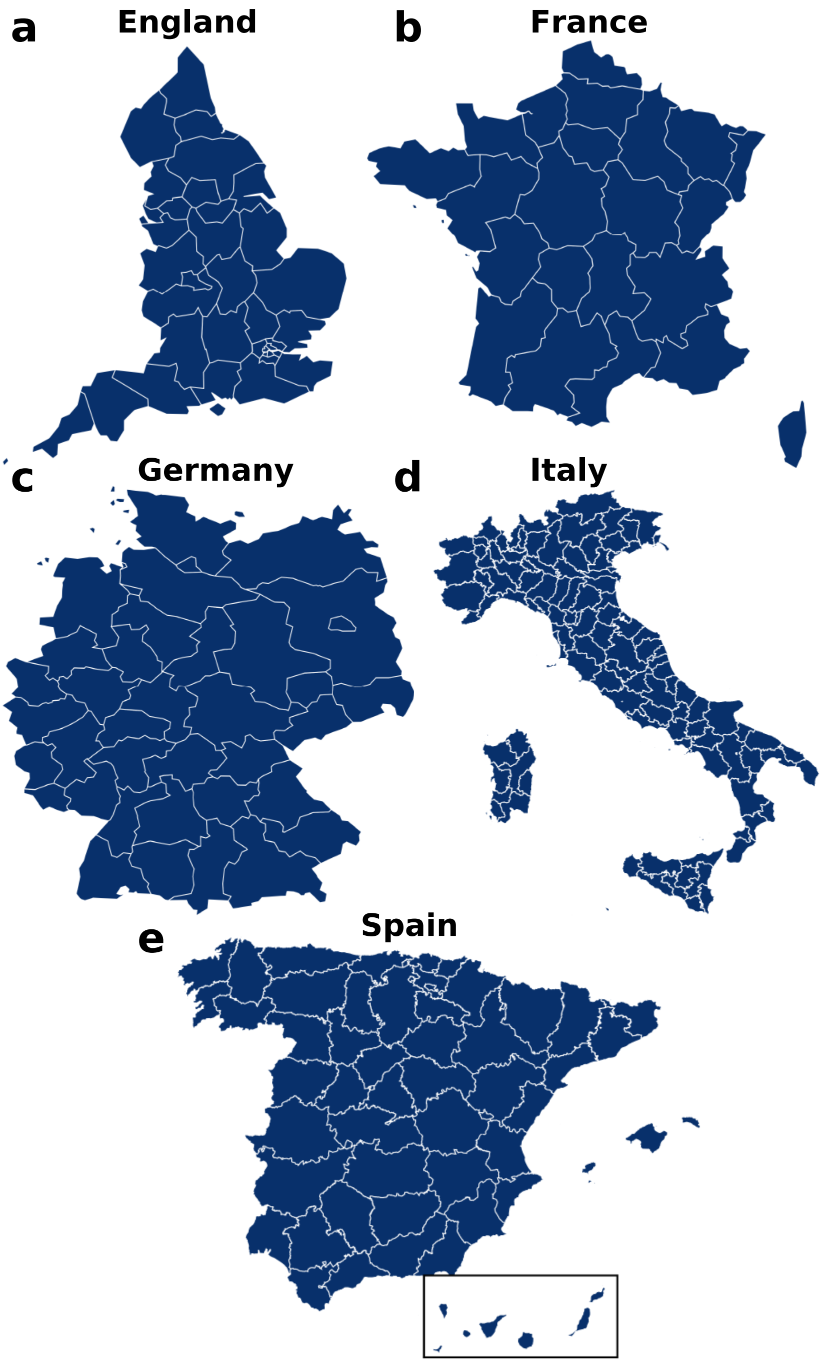


Fig A: **Local administrative subdivisions considered.** Covid and mobility data are aggregated at NUTS 2 level (regions or states) for England (**a**), France (**b**) and Germany (**c**), and at province (NUTS 3) level for Italy (**d**) and Spain (**e**). Administrative boundary data were obtained from GADM for Italy and Spain (`https://gadm.org`), and from EuroStat (`https://ec.europa.eu/eurostat`) for the rest of countries.

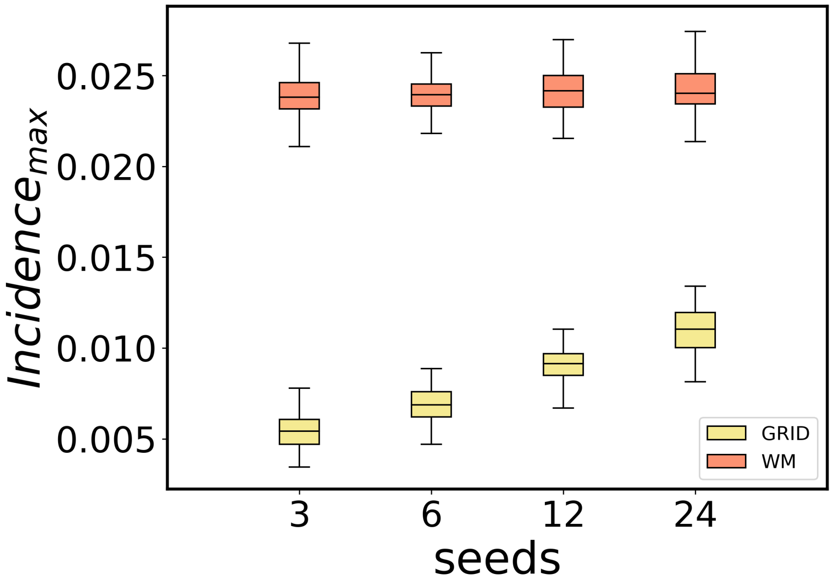


Fig B: **Incidence peaks boxplots for two populations simulation.** Distribution of incidence peaks as a function of imported seeds from the source for a gridded population in yellow and for a well-mixed population in orange.

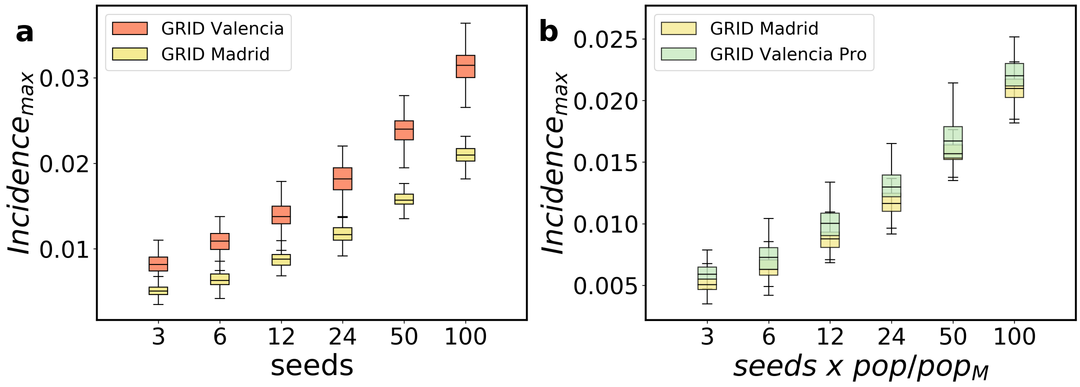


Fig C: **Incidence peaks dependence from seeds per capita.** Incidence peaks as a function of initial seeds in a single gridded population. **a** The same absolute number of initial seeds produces higher incidence peaks in a small population (Valencia) than in a bigger one (Madrid). **b** Setting the number of initial seeds in Valencia proportionally to the equivalent number of seeds per capita of Madrid, we get to similar incidence peaks.

| Province | Incidence onset | Incidence peak time |
|---|---|---|
| Alsace | 2020-03-11 | 2020-04-14 |
| Auvergne | 2020-03-23 | 2020-04-14 |
| Rhône-Alpes | 2020-03-16 | 2020-04-14 |
| Pays de la Loire | 2020-03-27 | 2020-04-08 |
| Poitou-Charentes | 2020-03-30 | 2020-04-14 |
| Limousin | 2020-03-28 | 2020-04-14 |
| Aquitaine | 2020-03-27 | 2020-04-21 |
| Picardie | 2020-03-15 | 2020-04-20 |
| Nord-Pas de Calais | 2020-03-26 | 2020-04-16 |
| Bourgogne | 2020-03-21 | 2020-04-23 |
| Franche-Comté | 2020-03-16 | 2020-04-15 |
| Provence-Alpes-Côte d'Azur | 2020-03-18 | 2020-04-16 |
| Mayotte | 2020-03-24 | 2020-04-03 |
| Champagne-Ardenne | 2020-03-22 | 2020-04-15 |
| Lorraine | 2020-03-15 | 2020-04-15 |
| Ile-de-France | 2020-03-20 | 2020-04-14 |
| Midi-Pyrénées | 2020-03-27 | 2020-04-14 |
| Languedoc-Roussillon | 2020-03-18 | 2020-04-07 |
| Bretagne | 2020-03-18 | 2020-03-29 |
| Centre - Val de Loire | 2020-03-21 | 2020-04-15 |
| Corse | 2020-03-10 | 2020-03-27 |
| Basse-Normandie | 2020-03-19 | 2020-04-12 |
| Haute-Normandie | 2020-03-21 | 2020-04-07 |
| La Réunion | nan | 2020-03-31 |
| Guyane | 2020-03-21 | 2020-03-29 |
| Martinique | 2020-03-27 | 2020-04-01 |
| Guadeloupe | 2020-03-19 | 2020-03-30 |

Table C: **Table of epidemic features for NUTS2 of France, when available.**

| Province | Incidence onset | Incidence peak time |
|---|---|---|
| Karlsruhe | 2020-03-12 | 2020-04-02 |
| Freiburg | 2020-03-11 | 2020-04-02 |
| Stuttgart | 2020-03-10 | 2020-03-26 |
| Berlin | 2020-03-11 | 2020-03-26 |
| Tübingen | 2020-03-11 | 2020-03-25 |
| Hannover | 2020-03-11 | 2020-03-25 |
| Lüneburg | 2020-03-15 | 2020-03-31 |
| Braunschweig | 2020-03-17 | 2020-03-27 |
| Weser-Ems | 2020-03-15 | 2020-03-26 |
| Schleswig-Holstein | 2020-03-16 | 2020-04-02 |
| Dresden | 2020-03-16 | 2020-03-26 |
| Leipzig | 2020-03-15 | 2020-04-02 |
| Chemnitz | 2020-03-16 | 2020-04-02 |
| Hamburg | 2020-03-10 | 2020-03-25 |
| Brandenburg | 2020-03-16 | 2020-04-08 |
| Rheinhessen-Pfalz | 2020-03-12 | 2020-04-02 |
| Trier | 2020-03-13 | 2020-03-28 |
| Koblenz | 2020-03-11 | 2020-03-26 |
| Schwaben | 2020-03-12 | 2020-04-02 |
| Unterfranken | 2020-03-12 | 2020-04-03 |
| Mittelfranken | 2020-03-17 | 2020-04-02 |
| Oberfranken | 2020-03-15 | 2020-04-03 |
| Oberpfalz | 2020-03-12 | 2020-04-02 |
| Niederbayern | 2020-03-12 | 2020-04-01 |
| Oberbayern | 2020-03-10 | 2020-03-26 |
| Mecklenburg-Vorpommern | 2020-03-17 | 2020-03-18 |
| Thüringen | 2020-03-17 | 2020-03-26 |
| Sachsen-Anhalt | 2020-03-17 | 2020-03-26 |
| Darmstadt | 2020-03-15 | 2020-03-26 |
| Gießen | 2020-03-15 | 2020-03-24 |
| Kassel | 2020-03-13 | 2020-04-02 |
| Bremen | 2020-03-10 | 2020-05-07 |
| Köln | 2020-03-05 | 2020-04-02 |
| Münster | 2020-03-12 | 2020-04-01 |
| Düsseldorf | 2020-03-12 | 2020-04-08 |
| Saarland | 2020-03-12 | 2020-04-01 |
| Detmold | 2020-03-11 | 2020-04-02 |
| Arnsberg | 2020-03-16 | 2020-04-02 |

Table D: **Table of epidemic features for NUTS2 of Germany, when available.**

| Province | Incidence onset | Incidence peak time | Province | Incidence onset | Incidence peak time |
|---|---|---|---|---|---|
| Torino | 2020-03-12 | 2020-04-16 | Perugia | 2020-03-14 | 2020-03-27 |
| Vercelli | 2020-03-11 | 2020-04-11 | Terni | 2020-03-09 | 2020-03-27 |
| Novara | 2020-03-12 | 2020-04-10 | Viterbo | 2020-03-17 | 2020-03-28 |
| Cuneo | 2020-03-14 | 2020-04-13 | Rieti | 2020-03-20 | 2020-03-31 |
| Asti | 2020-03-01 | 2020-04-21 | Roma | 2020-03-18 | 2020-03-22 |
| Alessandria | 2020-03-05 | 2020-04-05 | Latina | 2020-03-19 | 2020-03-21 |
| Aosta | 2020-03-06 | 2020-03-28 | Frosinone | 2020-03-21 | 2020-03-27 |
| Imperia | 2020-03-11 | 2020-04-06 | Caserta | nan | 2020-03-25 |
| Savona | 2020-02-27 | 2020-04-04 | Benevento | 2020-03-29 | 2020-03-31 |
| Genova | 2020-03-11 | 2020-04-07 | Napoli | 2020-03-20 | 2020-04-02 |
| La Spezia | 2020-03-08 | 2020-04-04 | Avellino | 2020-03-15 | 2020-04-02 |
| Varese | 2020-03-11 | 2020-03-28 | Salerno | 2020-03-27 | 2020-03-29 |
| Como | 2020-03-10 | 2020-04-12 | 'L"Aquila' | 2020-03-24 | 2020-04-03 |
| Sondrio | 2020-03-11 | 2020-04-13 | Teramo | 2020-03-16 | 2020-03-25 |
| Milano | 2020-03-06 | 2020-03-20 | Pescara | 2020-03-11 | 2020-03-20 |
| Bergamo | 2020-02-27 | 2020-03-21 | Chieti | 2020-03-18 | 2020-04-17 |
| Brescia | 2020-03-03 | 2020-03-19 | Campobasso | 2020-03-19 | 2020-05-09 |
| Pavia | 2020-02-29 | 2020-04-05 | Foggia | 2020-03-16 | 2020-03-31 |
| Cremona | 2020-02-27 | 2020-03-21 | Bari | 2020-03-18 | 2020-03-29 |
| Mantova | 2020-03-07 | 2020-03-20 | Taranto | 2020-03-27 | 2020-04-02 |
| Bolzano | 2020-03-10 | 2020-03-29 | Brindisi | 2020-03-14 | 2020-04-13 |
| Trento | 2020-03-09 | 2020-03-21 | Lecce | 2020-03-21 | 2020-03-31 |
| Verona | 2020-03-11 | 2020-04-10 | Potenza | 2020-03-18 | 2020-03-20 |
| Vicenza | 2020-03-11 | 2020-04-03 | Matera | 2020-03-23 | 2020-03-27 |
| Belluno | 2020-03-07 | 2020-04-20 | Cosenza | 2020-03-26 | 2020-04-17 |
| Treviso | 2020-03-10 | 2020-03-21 | Catanzaro | 2020-03-26 | 2020-03-28 |
| Venezia | 2020-03-07 | 2020-04-08 | Reggio di Calabria | 2020-03-18 | 2020-03-28 |
| Padova | 2020-03-01 | 2020-03-26 | Trapani | nan | 2020-04-05 |
| Rovigo | 2020-03-18 | 2020-04-22 | Palermo | 2020-03-23 | 2020-03-23 |
| Udine | 2020-03-12 | 2020-04-03 | Messina | 2020-03-20 | 2020-03-27 |
| Gorizia | 2020-03-12 | 2020-04-22 | Agrigento | 2020-03-29 | 2020-03-30 |
| Trieste | 2020-03-07 | 2020-04-17 | Caltanissetta | 2020-03-20 | 2020-04-04 |
| Piacenza | 2020-02-27 | 2020-03-19 | Enna | 2020-03-18 | 2020-03-26 |
| Parma | 2020-02-29 | 2020-03-23 | Catania | 2020-03-21 | 2020-03-22 |
| 'Reggio nell"Emilia' | 2020-03-07 | 2020-03-22 | Ragusa | 2020-03-26 | 2020-03-26 |
| Modena | 2020-03-07 | 2020-03-24 | Siracusa | 2020-03-25 | 2020-03-26 |
| Bologna | 2020-03-12 | 2020-03-28 | Sassari | 2020-03-14 | 2020-03-20 |
| Ferrara | 2020-03-16 | 2020-04-03 | Nuoro | 2020-03-10 | 2020-03-25 |
| Ravenna | 2020-03-12 | 2020-03-20 | Cagliari | 2020-04-03 | 2020-04-10 |
| Forlì-Cesena | 2020-03-12 | 2020-04-04 | Pordenone | 2020-03-12 | 2020-03-26 |
| Pesaro e Urbino | 2020-03-01 | 2020-03-14 | Isernia | 2020-03-27 | 2020-04-04 |
| Ancona | 2020-03-06 | 2020-03-21 | Oristano | 2020-04-19 | 2020-04-19 |
| Macerata | 2020-03-11 | 2020-03-23 | Biella | 2020-03-07 | 2020-03-21 |
| Ascoli Piceno | 2020-03-14 | 2020-03-27 | Lecco | 2020-03-06 | 2020-03-20 |
| Massa Carrara | 2020-03-06 | 2020-03-29 | Lodi | 2020-02-27 | 2020-03-04 |
| Lucca | 2020-03-12 | 2020-03-29 | Rimini | 2020-03-04 | 2020-03-22 |
| Pistoia | 2020-03-10 | 2020-03-19 | Prato | 2020-03-10 | 2020-03-31 |
| Firenze | 2020-03-13 | 2020-04-03 | Crotone | 2020-03-14 | 2020-03-20 |
| Livorno | 2020-03-13 | 2020-03-21 | Vibo Valentia | 2020-03-13 | 2020-03-27 |
| Pisa | 2020-03-13 | 2020-03-29 | Verbano-Cusio-Ossola | 2020-03-12 | 2020-04-07 |
| Arezzo | 2020-03-14 | 2020-03-17 | Monza e della Brianza | 2020-03-11 | 2020-03-20 |
| Siena | 2020-03-13 | 2020-03-29 | Fermo | 2020-03-13 | 2020-03-26 |
| Grosseto | 2020-03-13 | 2020-03-27 | Barletta-Andria-Trani | 2020-03-22 | 2020-04-11 |
| | | | Sud Sardegna | 2020-03-26 | 2020-03-27 |

Table E: **Table of epidemic features for provinces of Italy, when available.**

| Province | Incidence onset | Incidence peak time | Mortality onset | Mortality peak time |
|---|---|---|---|---|
| Álava | 2020-03-07 | 2020-03-25 | 2020-03-16 | 2020-03-31 |
| Albacete | 2020-03-17 | 2020-03-29 | 2020-03-17 | 2020-04-05 |
| Alicante | 2020-03-24 | 2020-03-29 | 2020-03-21 | 2020-04-03 |
| Almería | nan | 2020-03-28 | 2020-03-22 | 2020-04-10 |
| Ávila | 2020-03-18 | 2020-03-27 | 2020-03-17 | 2020-03-26 |
| Badajoz | 2020-03-25 | 2020-03-27 | 2020-03-24 | 2020-04-09 |
| Baleares | 2020-03-22 | 2020-03-26 | 2020-03-21 | 2020-04-01 |
| Barcelona | 2020-03-15 | 2020-03-25 | 2020-03-12 | 2020-03-31 |
| Burgos | 2020-03-19 | 2020-03-24 | 2020-03-16 | 2020-03-31 |
| Cáceres | 2020-03-19 | 2020-03-27 | 2020-03-14 | 2020-04-01 |
| Cádiz | nan | 2020-04-01 | 2020-03-27 | 2020-04-08 |
| Castellón | 2020-03-23 | 2020-03-26 | 2020-03-21 | 2020-04-08 |
| Ciudad Real | 2020-03-17 | 2020-04-04 | 2020-03-17 | 2020-04-09 |
| Córdoba | 2020-03-28 | 2020-04-04 | 2020-03-19 | 2020-03-31 |
| A Coruña | 2020-03-22 | 2020-04-01 | 2020-03-22 | 2020-04-01 |
| Cuenca | 2020-03-19 | 2020-04-08 | 2020-03-17 | 2020-03-27 |
| Girona | 2020-03-18 | 2020-03-25 | 2020-03-17 | 2020-04-03 |
| Granada | 2020-03-23 | 2020-03-30 | 2020-03-16 | 2020-03-28 |
| Guadalajara | 2020-03-16 | 2020-03-31 | 2020-03-21 | 2020-03-25 |
| Guipúzcoa | 2020-03-22 | 2020-03-25 | 2020-03-20 | 2020-04-10 |
| Huelva | nan | 2020-04-02 | 2020-03-27 | 2020-04-06 |
| Huesca | 2020-03-23 | 2020-04-01 | 2020-03-21 | 2020-04-09 |
| Jaén | 2020-03-23 | 2020-03-30 | 2020-03-13 | 2020-04-08 |
| León | 2020-03-22 | 2020-03-28 | 2020-03-16 | 2020-04-03 |
| Lleida | 2020-03-18 | 2020-03-25 | 2020-03-16 | 2020-03-26 |
| La Rioja | 2020-03-07 | 2020-03-27 | 2020-03-11 | 2020-04-06 |
| Lugo | 2020-03-25 | 2020-04-01 | 2020-03-23 | 2020-04-01 |
| Madrid | 2020-03-09 | 2020-03-25 | 2020-03-09 | 2020-03-27 |
| Málaga | 2020-03-24 | 2020-03-24 | 2020-03-14 | 2020-04-10 |
| Murcia | 2020-03-25 | 2020-03-25 | 2020-03-24 | 2020-04-05 |
| Navarra | 2020-03-14 | 2020-03-26 | 2020-03-15 | 2020-03-30 |
| Ourense | 2020-03-22 | 2020-04-01 | 2020-03-22 | 2020-04-01 |
| Asturias | 2020-03-18 | 2020-03-19 | 2020-03-19 | 2020-04-10 |
| Palencia | 2020-03-20 | 2020-03-27 | 2020-03-24 | 2020-04-04 |
| Las Palmas | nan | 2020-03-28 | 2020-03-20 | 2020-03-21 |
| Pontevedra | 2020-03-21 | 2020-04-01 | 2020-03-26 | 2020-03-31 |
| Salamanca | 2020-03-17 | 2020-03-28 | 2020-03-16 | 2020-03-28 |
| Santa Cruz de Tenerife | 2020-03-24 | 2020-03-26 | 2020-03-20 | 2020-03-21 |
| Cantabria | 2020-03-19 | 2020-03-26 | 2020-03-20 | 2020-03-31 |
| Segovia | 2020-03-17 | 2020-04-07 | 2020-03-16 | 2020-03-30 |
| Sevilla | nan | 2020-03-28 | 2020-03-22 | 2020-04-07 |
| Soria | 2020-03-17 | 2020-03-25 | 2020-03-16 | 2020-03-30 |
| Tarragona | 2020-03-24 | 2020-03-31 | 2020-03-21 | 2020-04-08 |
| Teruel | 2020-03-23 | 2020-03-26 | 2020-03-16 | 2020-04-01 |
| Toledo | 2020-03-17 | 2020-03-26 | 2020-03-17 | 2020-04-09 |
| Valencia | 2020-03-23 | 2020-03-24 | 2020-03-21 | 2020-04-02 |
| Valladolid | 2020-03-19 | 2020-04-10 | 2020-03-18 | 2020-04-01 |
| Vizcaya | 2020-03-19 | 2020-03-25 | 2020-03-17 | 2020-04-08 |
| Zamora | 2020-03-22 | 2020-04-03 | 2020-03-17 | 2020-04-03 |
| Zaragoza | 2020-03-23 | 2020-03-26 | 2020-03-10 | 2020-04-01 |
| Ceuta | 2020-03-30 | 2020-03-30 | 2020-03-26 | 2020-04-05 |
| Melilla | 2020-04-03 | 2020-04-03 | 2020-03-24 | 2020-03-24 |

Table F: **Table of epidemic features for provinces of Spain, when available.**

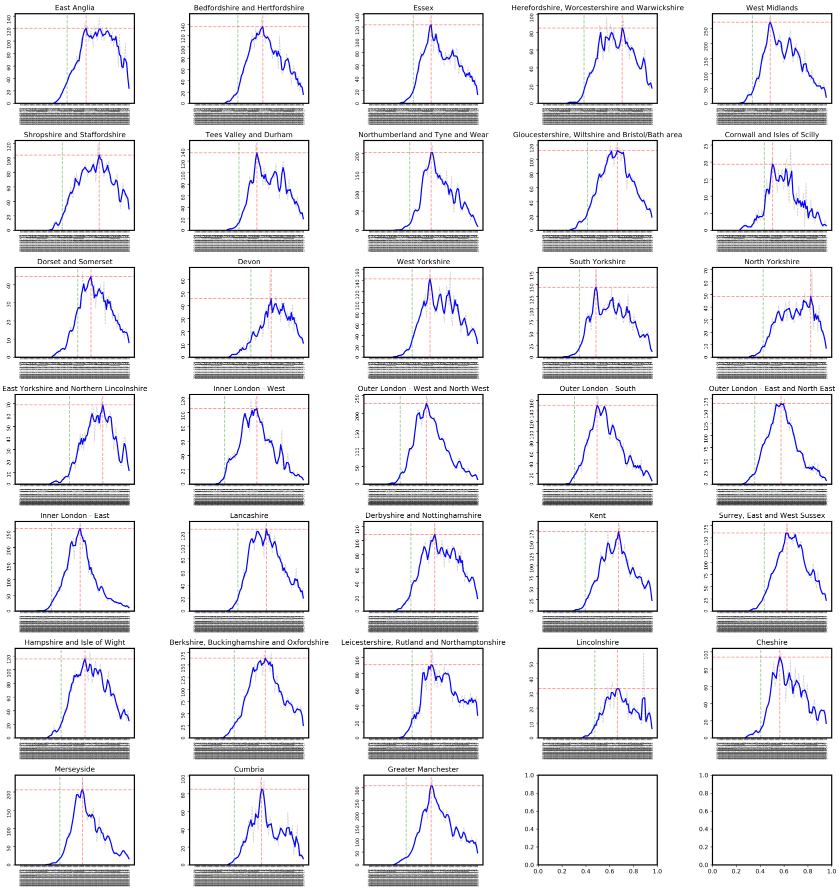


Fig D: **Epidemic curves of confirmed cases for the NUTS2 of England.** Green dashed line to represent the incidence onset, red dashed line to represent the incidence peak time.

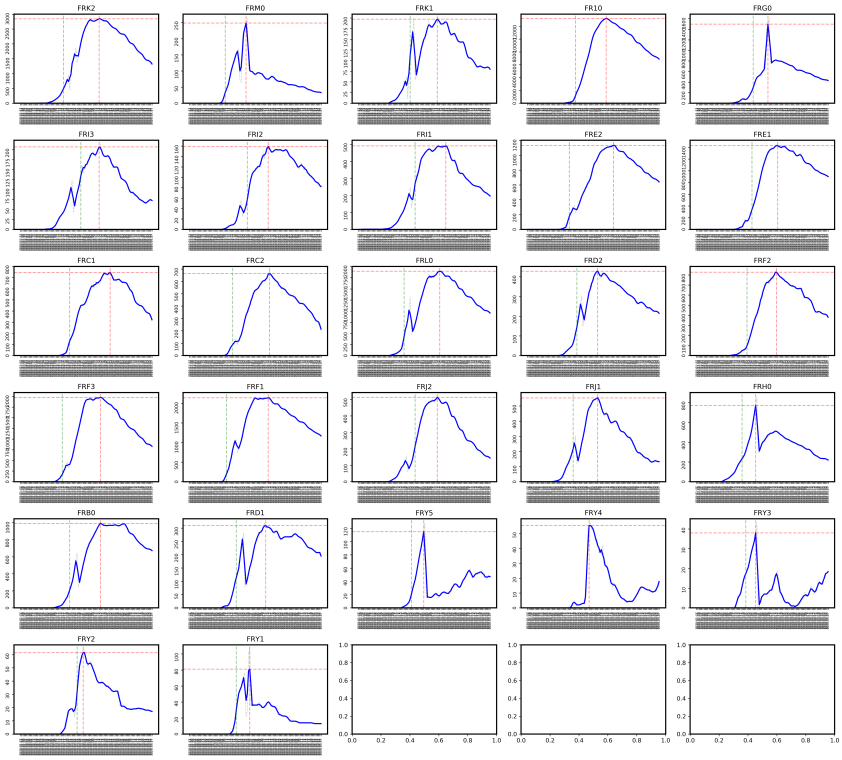


Fig E: **Epidemic curves of confirmed cases for the NUTS2 of France.** Green dashed line to represent the incidence onset, red dashed line to represent the incidence peak time.

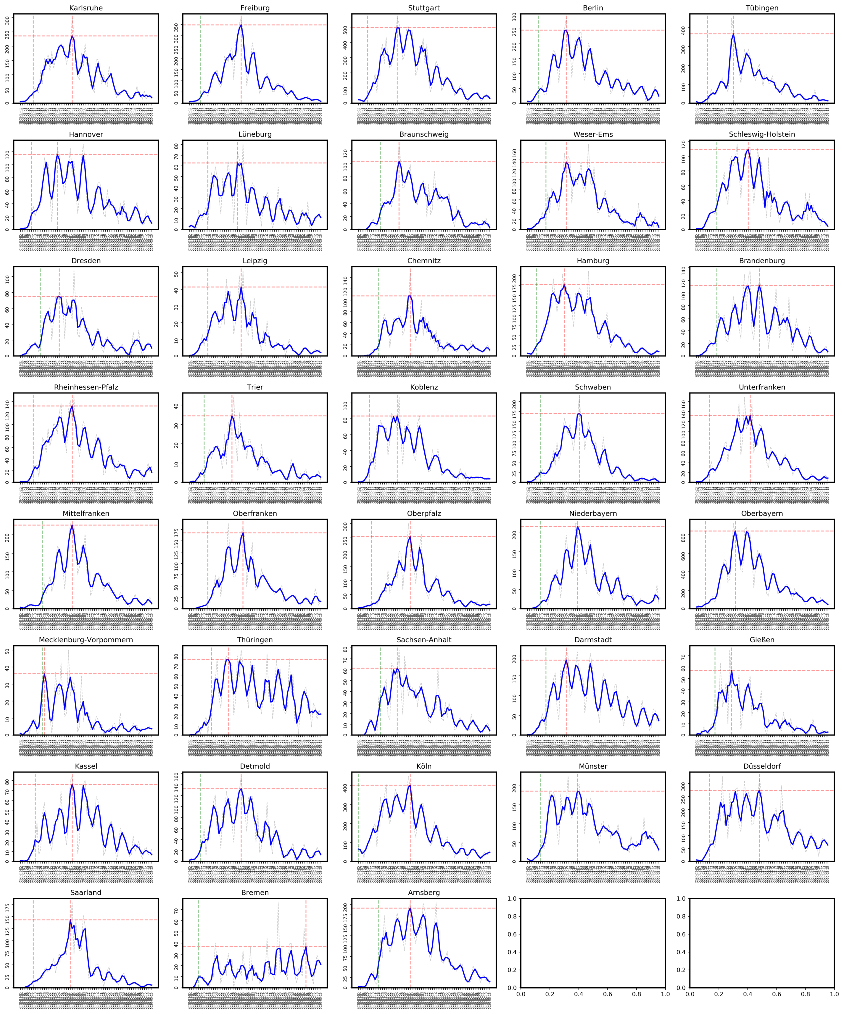


Fig F: **Epidemic curves of confirmed cases for the NUTS2 of Germany.** Green dashed line to represent the incidence onset, red dashed line to represent the incidence peak time.

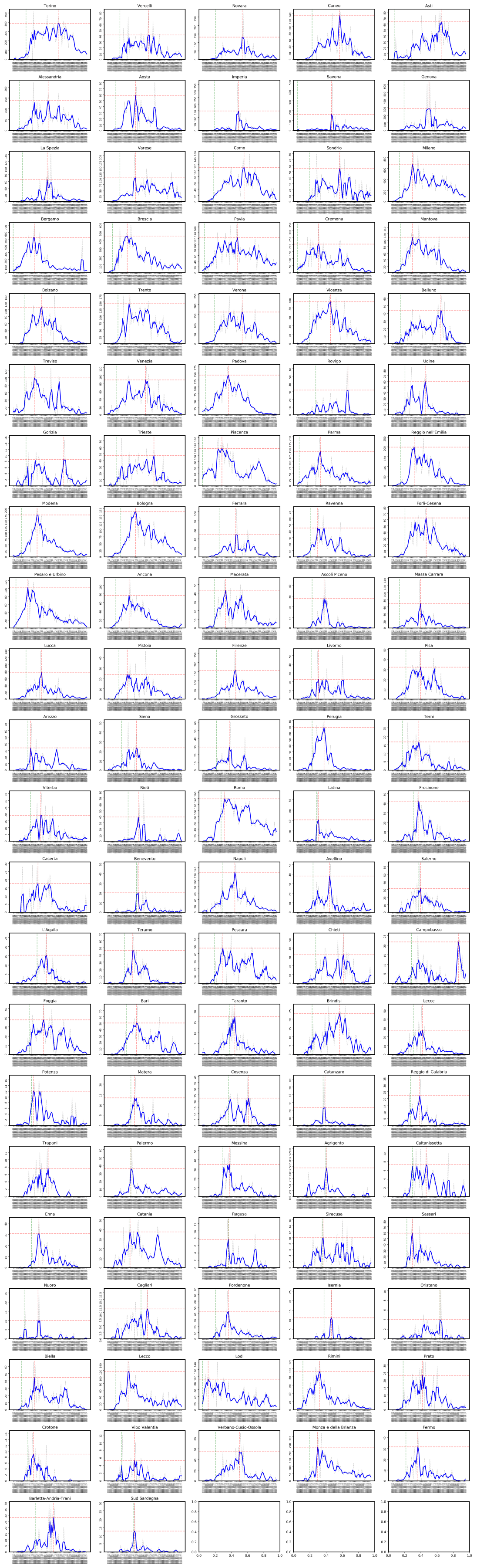


Fig G: **Epidemic curves of confirmed cases for the NUTS2 of Italy.** Green dashed line to represent the incidence onset, red dashed line to represent the incidence peak time.

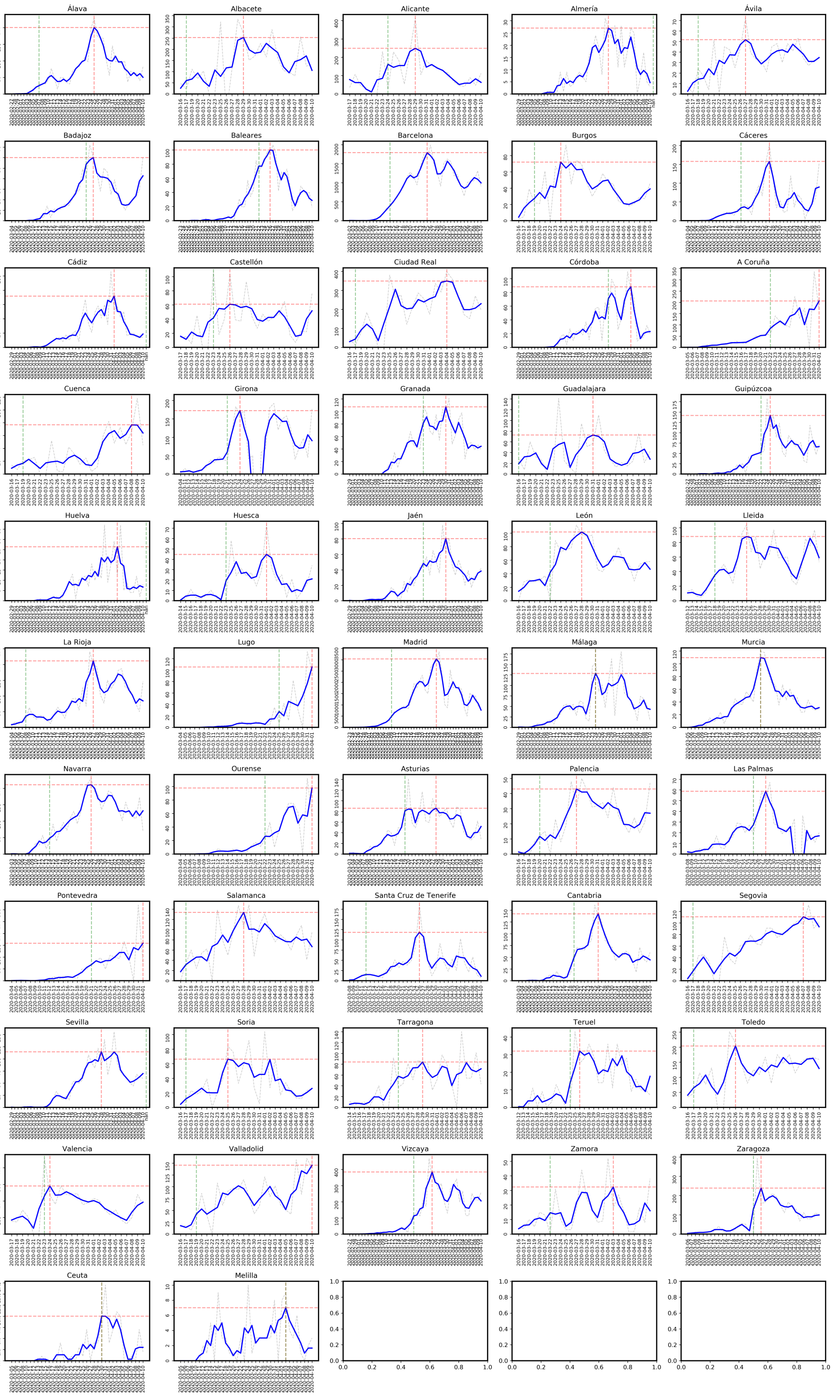


Fig H: **Epidemic curves of confirmed cases for provinces of Spain.** Green dashed line to represent the incidence onset, red dashed line to represent the incidence peak time.

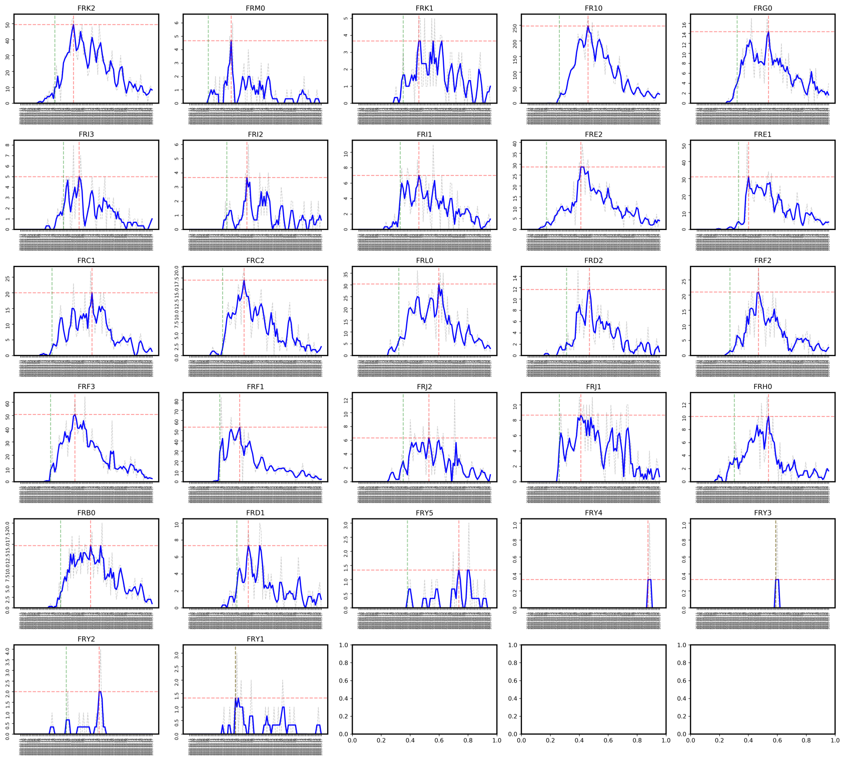


Fig I: **Epidemic curves of reported deaths for NUTS2 of France.** Green dashed line to represent the mortality onset, red dashed line to represent the mortality peak time.

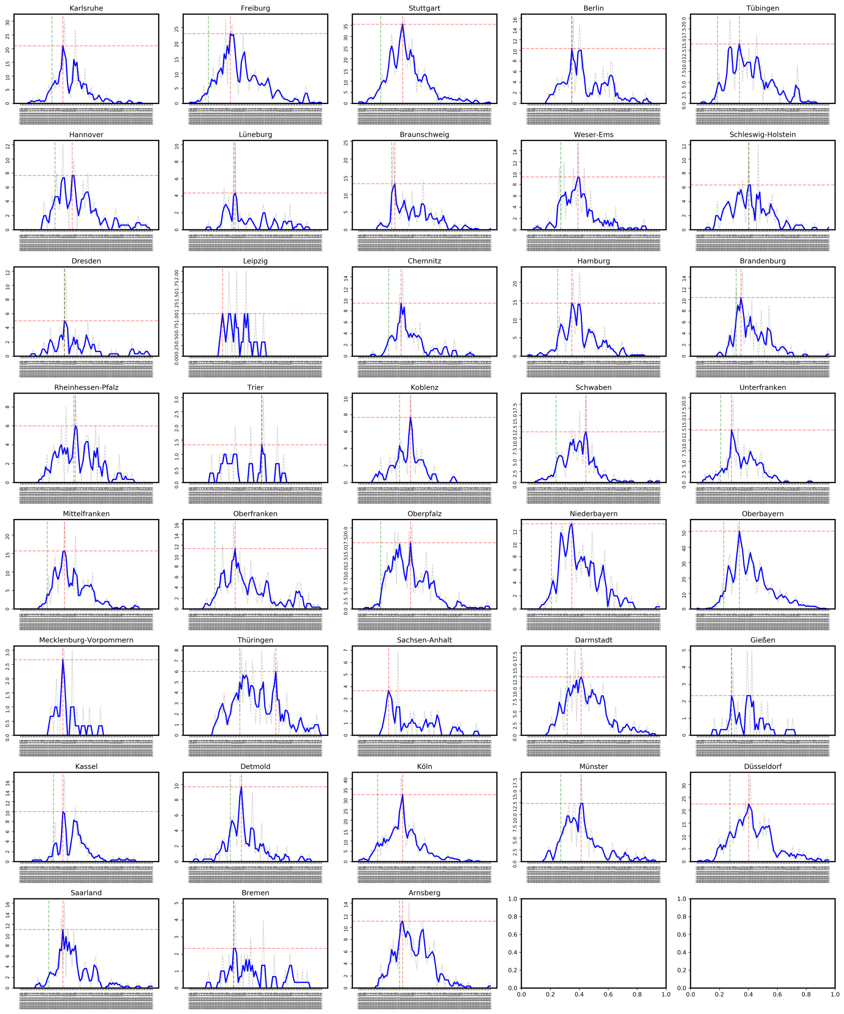


Fig J: **Epidemic curves of reported deaths for NUTS2 of Germany.** Green dashed line to represent the mortality onset, red dashed line to represent the mortality peak time.

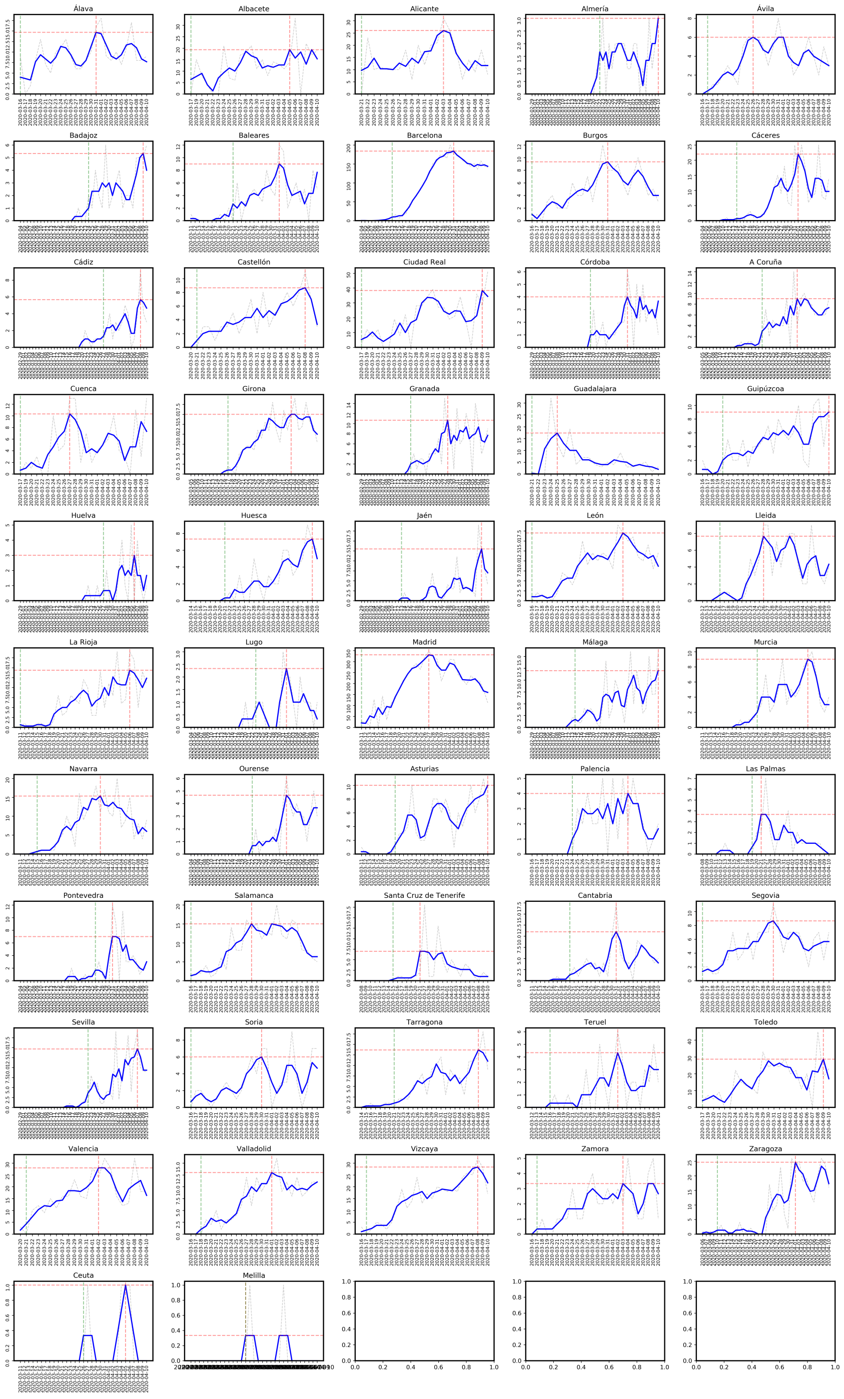


Fig K: **Epidemic curves of reported deaths for provinces of Spain.** Green dashed line to represent the mortality onset, red dashed line to represent the mortality peak time.

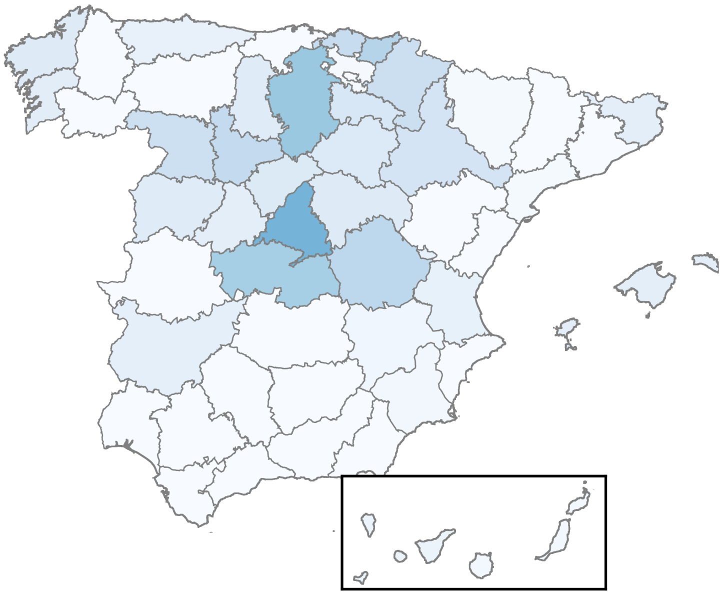

Fig L: **Corrected correlation** $R^*$ **for each area of origin in Spain with Cuebiq data.** The outcome scenario looks consistent with the same figure obtained from mobile phone data. Administrative boundary data were obtained from GADM for Spain (`https://gadm.org`).

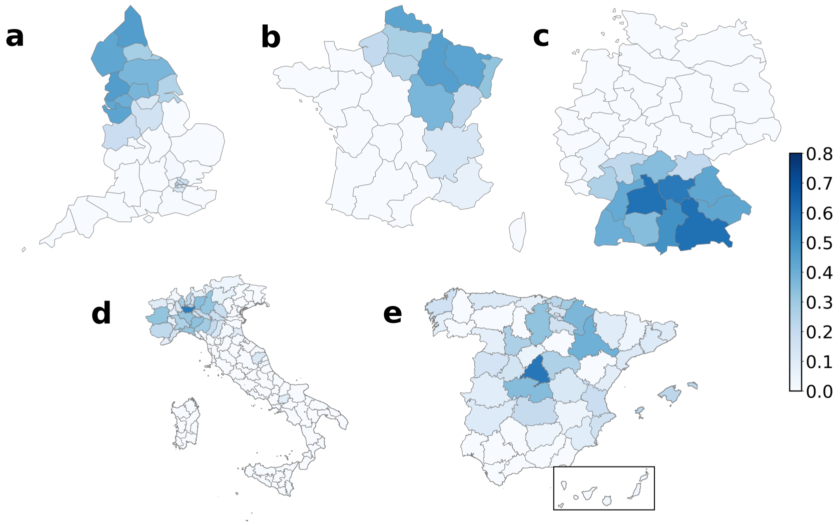


Fig M: **Origin analysis reversed map**. Conversely to the analysis in the main paper, for each destination of each country we check the corrected R-squared $R^*$ correlation of mobility and origin incidence peaks. Darker areas represent those areas where most probably the spread was originated from. Administrative boundary data were obtained from GADM for Italy and Spain (`https://gadm.org`), and from EuroStat (`https://ec.europa.eu/eurostat`) for the rest of countries.

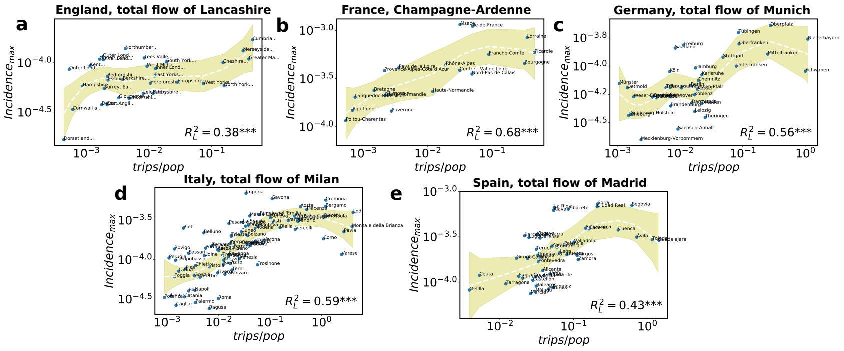


Fig N: **Correlations of incidence peaks and total flows.** Peak of incidence versus total trips per local capita received and started from **a** Lancashire, **b** Champagne-Ardenne, **c** Munich, **d** Milan, **e** Madrid during two weeks until one week before the local onset. In all cases, the curves with the fits are obtained using the non parametric LOESS method, as also the values of $R^2_L$. The shaded areas correspond to the 95% confidence interval.

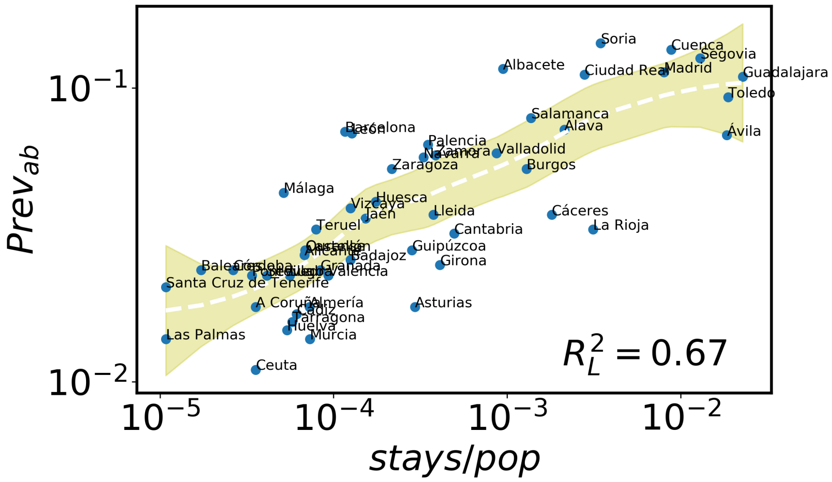


Fig O: **Stays per capita versus temporal size at May 13th 2020.** Correlation plot of stays per capita of weekenders from Madrid to other provinces versus size of prevalence in the local population.

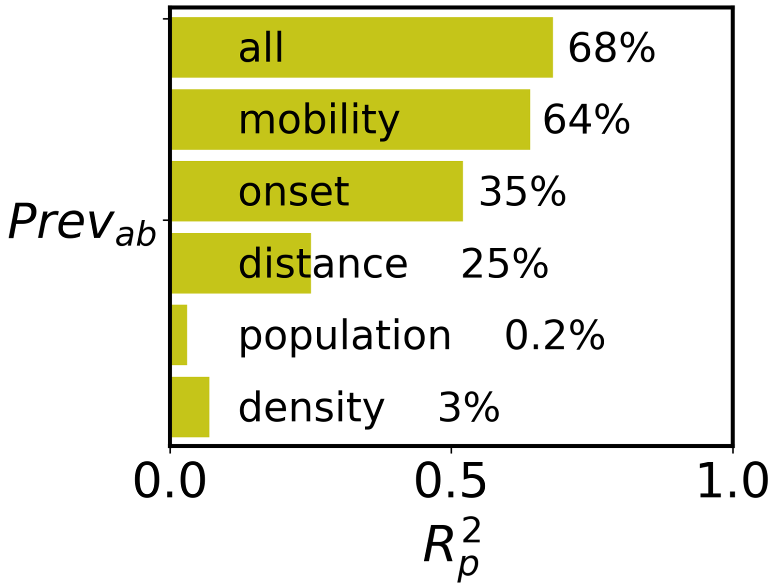


Fig P: **Correlation analysis of antibodies prevalence at May 13th 2020.** Variance of local size at May 13th 2020 explained by mobility, population, population density and distance from Madrid of each province.

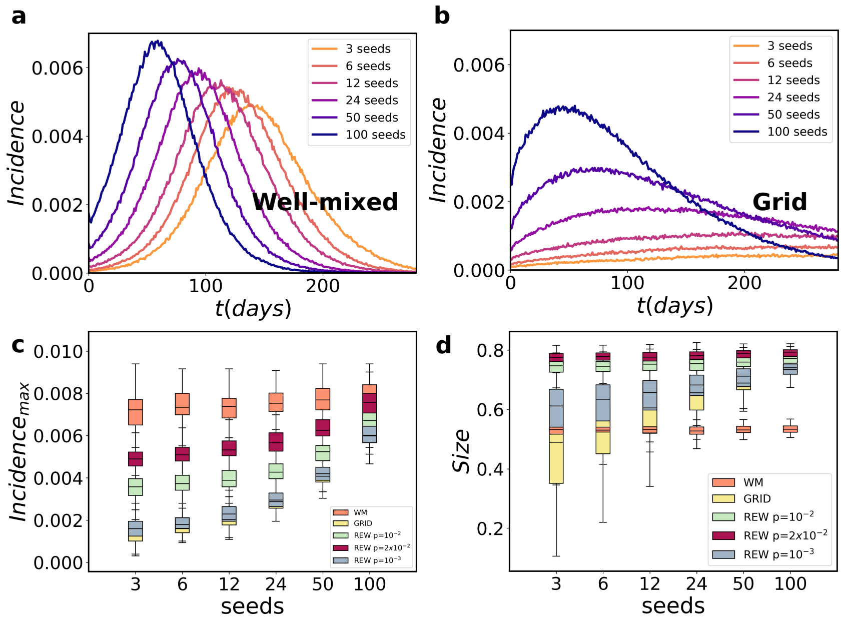


Fig Q: **Model and multi-seeding in a single population.** Average incidence curves versus number of seeds in a single population with **a** the well-mixed case and **b** the GRID topology. **c** Box-plot of the distribution of incidence peaks and **d** final epidemic sizes for a well-mixed (WM), the grid (GRID) and the rewired grid (REW) with different rewiring probability p as a function of the number of seeds. One hundred simulations have been run for each scenario. $\beta = 0.12$ days$^{-1}$ for the well-mixed case and $\beta = 0.31$ days$^{-1}$ for the gridded contact network.

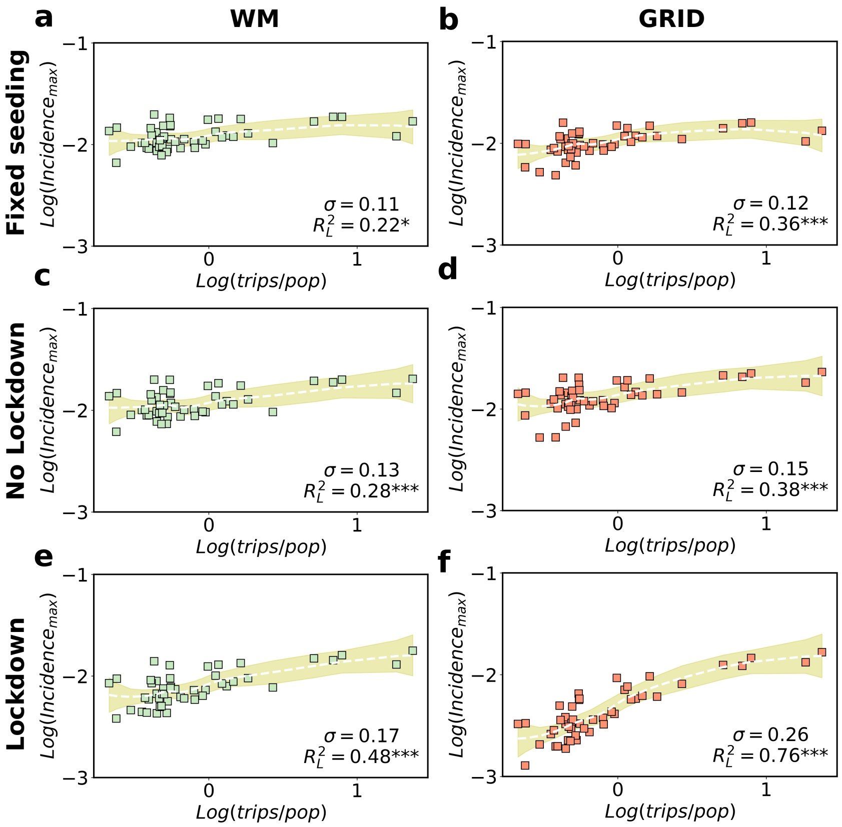


Fig R: **Models with heterogeneous populations.** Comparison of the effects of confinement policies, seeding and topology on the correlation between incidence peaks and mobility from the source (seeding) in a metapopulation model. In all the plots the height of the incidence peaks are displayed as a function of the incoming trips in every province divided by the local population. In **a**, simulation of well-mixed populations in each province allowing to travel only a fixed number of seeds from the source (Madrid). In **b**, the same for gridded contact networks in the provinces. Both scenarios are again considered in **c** and **d**, but this time without constraints in the travels between provinces and allowing thus multi-seeding. Finally, in **e** and **f**, the simulations are repeated with a national lockdown applied when Madrid arrives at 2000 cases. All results are averaged over 100 simulations with each square corresponding to the average value obtained in a province. $\beta = 0.12$ days$^{-1}$ for the well-mixed case and $\beta = 0.31$ days$^{-1}$ for the gridded contact network.

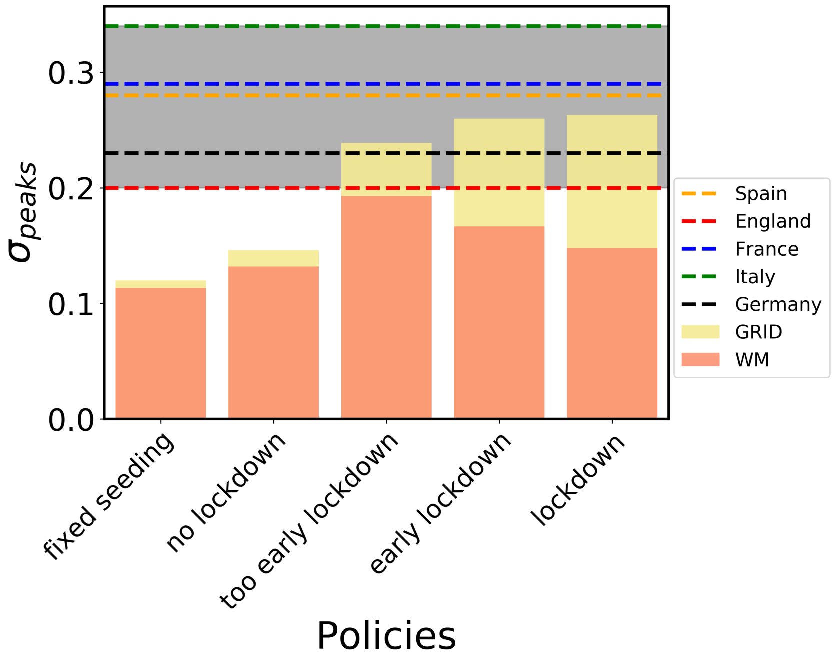


Fig S: **Policy comparison.** Incidence peaks standard deviation in for the multipopulation system with different interventions scenarios. The standard deviation is measured in plots similar to those shown in Fig. 4 both for empirical data and for the model simulations. The number of seeds in the empirical data is related to the mobility, while in the fixed seeding simulations we let each subpopulation reach a common threshold of cumulative incidence before closing the province borders. No lockdown is the scenario with no interventions. Too early, early and normal lockdown corresponds to a national lockdowns issued when Madrid (the source) reaches 1000, 2000 and 3000 total cases, respectively. After the lockdown $\beta$ gets halved and all trips across borders stop. $\beta = 0.12$ days$^{-1}$ for the well-mixed case and $\beta = 0.31$ days$^{-1}$ for the gridded contact network.

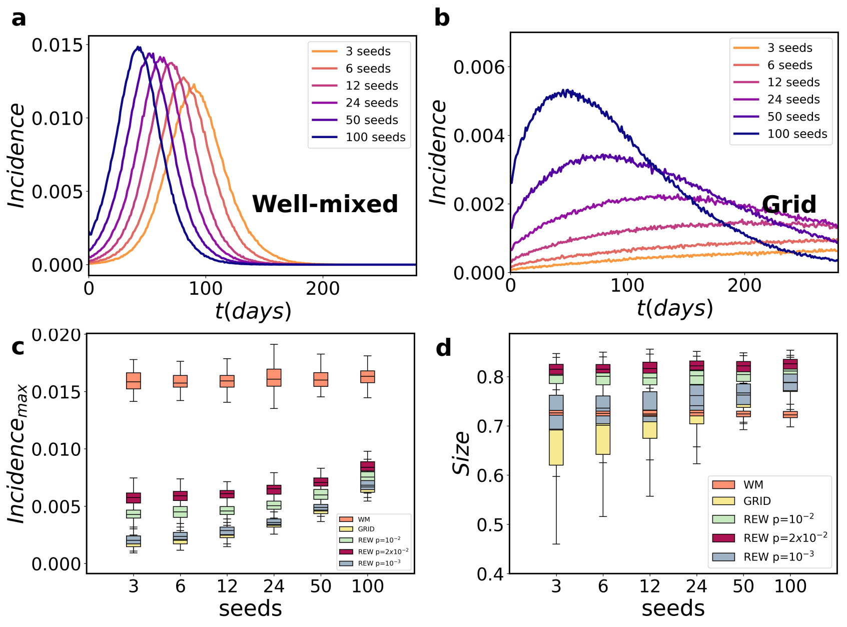


Fig T: **Model and multi-seeding in a single population.** Average incidence curves versus number of seeds in a single population with **a** the well-mixed case and **b** the GRID topology. **c** Box-plot of the distribution of incidence peaks and **d** final epidemic sizes for a well-mixed (WM), the grid (GRID) and the rewired grid (REW) with different rewiring probability p as a function of the number of seeds. One hundred simulations have been run for each scenario. $\beta = 0.15$ days$^{-1}$ for the well-mixed case and $\beta = 0.33$ days$^{-1}$ for the gridded contact network.

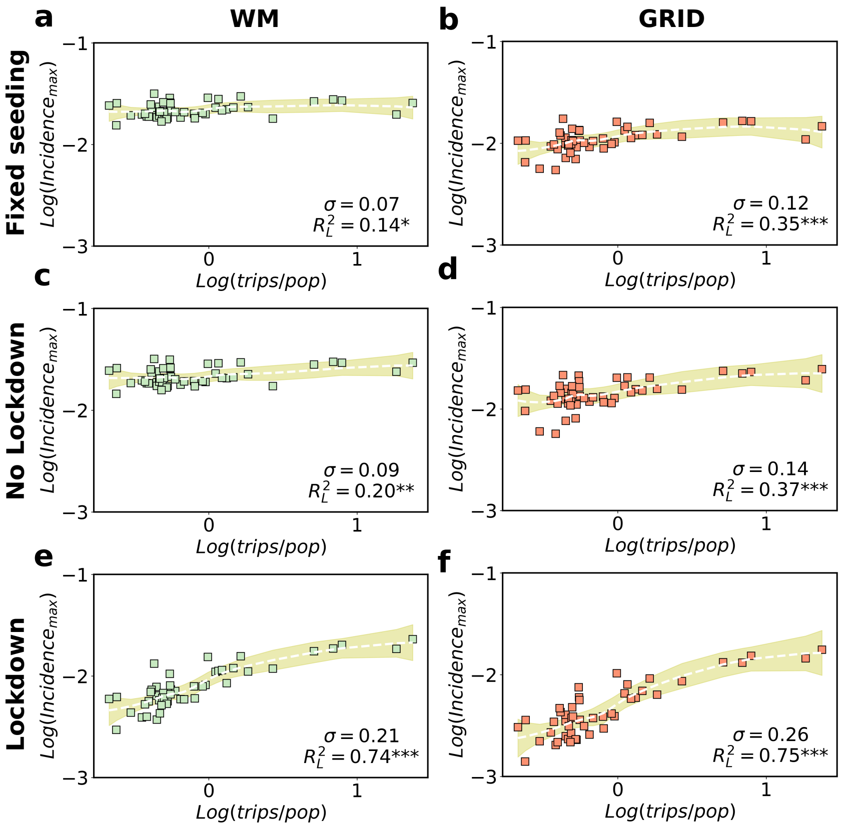


Fig U: **Models with heterogeneous populations.** Comparison of the effects of confinement policies, seeding and topology on the correlation between incidence peaks and mobility from the source (seeding) in a metapopulation model. In all the plots the height of the incidence peaks are displayed as a function of the incoming trips in every province divided by the local population. In **a**, simulation of well-mixed populations in each province allowing to travel only a fixed number of seeds from the source (Madrid). In **b**, the same for gridded contact networks in the provinces. Both scenarios are again considered in **c** and **d**, but this time without constraints in the travels between provinces and allowing thus multi-seeding. Finally, in **e** and **f**, the simulations are repeated with a national lockdown applied when Madrid arrives at 2000 cases. All results are averaged over 100 simulations with each square corresponding to the average value obtained in a province. $\beta = 0.15$ days$^{-1}$ for the well-mixed case and $\beta = 0.33$ days$^{-1}$ for the gridded contact network.

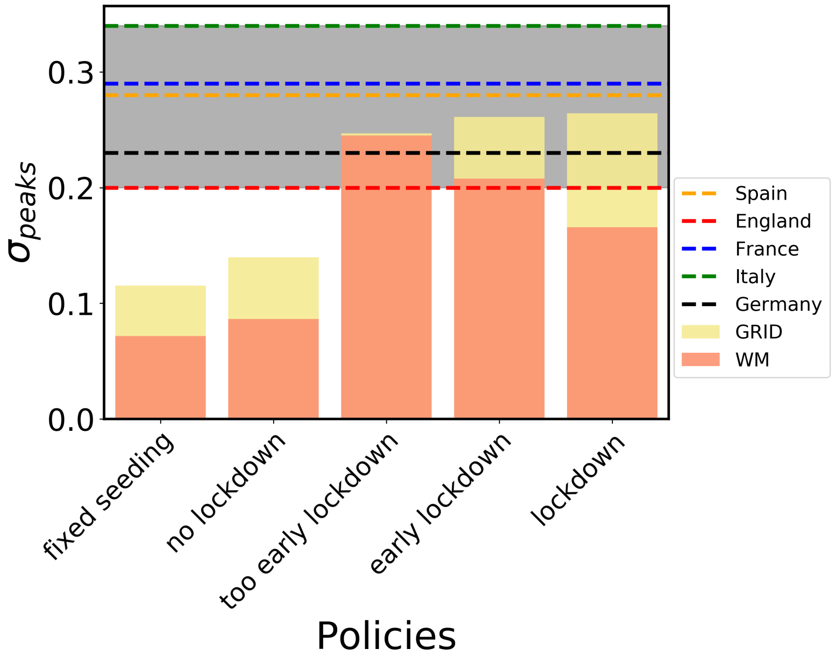


Fig V: **Policy comparison.** Incidence peaks standard deviation for the multipopulation system with different interventions scenarios. The standard deviation is measured in plots similar to those shown in Fig. 4 both for empirical data and for the model simulations. The number of seeds in the empirical data is related to the mobility, while in the fixed seeding simulations we let each subpopulation reach a common threshold of cumulative incidence before closing the province borders. No lockdown is the scenario with no interventions. Too early, early and normal lockdown corresponds to a national lockdowns issued when Madrid (the source) reaches 1000, 2000 and 3000 total cases, respectively. After the lockdown $\beta$ gets halved and all trips across borders stop. $\beta = 0.15$ days$^{-1}$ for the well-mixed case and $\beta = 0.33$ days$^{-1}$ for the gridded contact network.

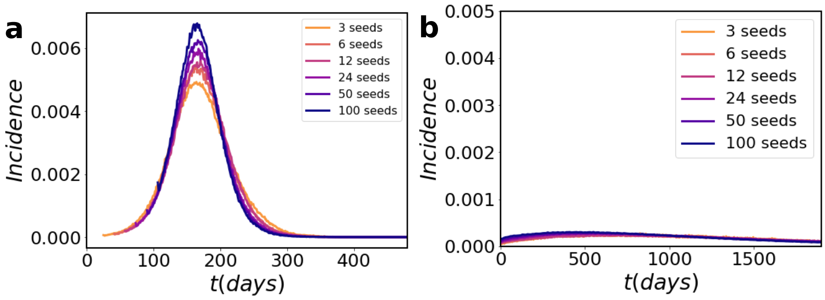


Fig W: **Collapse of the epidemic curves in a single population for lower $\beta$.** The collapse of the epidemic curves produced by a different number of initial seeds in a WM population in **a** and in a population with a GRID contact network in **b**. The peak time obeys to a power-law rule in the GRID scenario, while in the WM scenario it shows a logatithmic dependence on the seeds. $\beta = 0.12$ days$^{-1}$ for the well-mixed case and $\beta = 0.31$ days$^{-1}$ for the gridded contact network.
