# Supplementary material for: Interplay between mobility, multi-seeding and lockdowns shapes COVID-19 local impact
Source: PLoS Comput Biol. 2021 Oct 14;17(10):e1009326. doi: 10.1371/journal.pcbi.1009326 (PMC8516261; doi:10.1371/journal.pcbi.1009326)
Supplement: Attachment — Submitted filename: response-reviewers.pdf [file pcbi.1009326.s002.pdf]

Response to Reviewers' Questions

Reviewer #1:

In their current manuscript, the authors investigate spreading dynamics described by a compartmental model on a network. In particular, they consider the impact of simultaneous importation (seeding) of infected and the implications of non-pharmaceutical interventions such as lockdowns and travel restrictions. The study is inspired by the current SARS-CoV-2 pandemic. The underlying network that forms the substrate of interactions is derived from mobility data (mobile-phone traces) of 5 European countries: Spain, England, France, Germany, and Italy. The main finding is that parallel seeding and interventions lead to spatially heterogeneous outbreak patterns (opposed to a diffusion wave). The study focuses on the period of the first wave in spring 2020.

The considered model of the spreading is taken from previous literature. Therefore, the novelty arises from the inclusion of empirical data and multiple importations. Due to modern mobility behavior, countries are closely linked to each other and any globally acting virus will have many different entry points into a country. The main points of my criticism concern the following questions: Why now? How innovative?

Why now?

The period considered in the study is spring 2020, when the first wave of the current pandemic occurred. Since then, there have been 2 or more additional waves. That triggers the question how universal the findings are. Similarly, how relevant are the findings in light of vaccination campaigns that have gathered speed in the countries considered?

First of all, we would like to thank the reviewer for the insightful comments. Indeed, the novelty of this work is not in the models, which have been considered before. The novelty stands in the so far neglected effect of the arrival of multiple seeds on the local outbreak severity. Previous studies considered the effect of the fraction of mobility out-flow from epidemic origins to the rest of the world, however they focused exclusively on the effect on arrival times and early stages of the outbreaks. Here, we analyze the effect of mobility on later stages, which uncovers an important relation between early incoming mobility from the origin, hence multiseeding, on the subsequent local propagation of a disease, which unleashes clear non-linear and non-trivial effects.

We decided to focus on the first wave of the pandemic because during this period the population is fully susceptible and thus the impact of the arrival of new seeds can be studied in a cleaner way. After the first wave, which stopped thanks to the mitigation measures, there is a seroprevalence background in the population due to the individuals who have recovered from the disease, which is rather heterogeneous in space and that would difficult the analysis of multiseeding impacts. This is why we selected the first wave, since the propagation patterns were easy to characterize and, therefore, multiseeding could be associated to mobility from one or a few geographical sources. However, a generalization to subsequent waves can be implemented as long as the level of already recovered individuals is considered in the model. The level of information required is not trivial, but it is not impossible. This phenomenon is important especially in the light of new variants that, with a non-negligible probability, are able to escape the immunity provided by vaccinations and wild type seroprevalence.

Regarding the opportunity, and why now, there are previous works mentioning only from a theoretical perspective, mainly in a single population, that multiseeding should play a role in spreading. We have performed a systematic analysis in a metapopulation context from a theoretical perspective, but, not only that, we have found serious indications of how multiseeding increases peaks of incidence, final sizes and, consequently, mortality in empirical data of COVID-19 across Europe. Please note that with all the cautions on quality, the extension and detail of COVID-19 data is unprecedented in epidemic history.

These findings may be relevant to geographically design new vaccination campaigns for new variants, e.g. booster shots campaigns. Policies should take into account the spatial heterogeneity in seroprevalence of new strains (such as the Gamma and Delta variants, which show lower vaccine efficacies and low values of cross-immunity induced by wild type infections) and mobility from places already infected by the variant.

We have updated the introduction to leave these points clearer.

How innovative?

The observation that an increased number of importations of infected leads to increased numbers of infected is almost self-explanatory. Every infected arriving at a susceptible part of populations acts as an independent nucleation core of a local outbreak.

Indeed, every arriving seed acts as an independent (in very first approximation) nucleation core of a local outbreak. However, the effects of the seed arrival across the social network are felt well within the

development of the epidemic wave and they are far from trivial. Beyond the more-more simple relation, the multiseeding affects the speed of the spreading, the height of peaks and the final size. Not only that, the important variable is not the raw number of seeds but the ratio of seed number over local population.

We offer a complete and quantitative characterization of a phenomenon that has seldom been studied before. In fact, the effect of multiseeding is negligible when considering homogeneously mixed populations as done in the vast majority of metapopulation models currently in the literature. For these reasons, this non-linear relationship is impossible to study in classical frameworks. However, real contact networks always exhibit a spatial structure, at least, at the smallest scale. Our point here is that adding this spatial ingredient allow us to quantify this effect that is seen in the data. The results can be easily seen, for example, in Fig 1a-b for a single population model. In this scenario, doubling the number of imported cases leads to a non-linear effect both on the incidence peaks and in the speed of spreading in the population after the early stage.

These points have been discussed with more detail now in the introduction and in the conclusions.

Concerning the inclusion of mobile-phone data, I agree that these data sets have a lot to offer in terms of temporal and spatial granularity. On the other hand, what is the advantage compared to census data from pre-COVID times adjusted for movement restrictions (or other data sets such as Google mobility)?

The question is very significant and much needed to understand the great importance of mobile phone data to inform public health policies (`https://advances.sciencemag.org/content/6/23/eabc0764`). Census data usually offers commuting mobility, which represents a little more than one half of mobility, but it does not provide the whole picture. Furthermore, mobility is not constant over all the days of the week, whereas censuses have only one register in most of the cases for every decade. In a previous work we analyzed the effect on spreading of two different types of mobility in Spain and people who usually moved on weekends resulted to be more influential than people traveling on weekdays, Ref. [27]. If the purpose of the study is to understand the role of all the mobility on epidemic spreading at inter-urban level, commuting flows alone are not a suitable choice since, independently of how they are modulated, they do not originally include all the mobility.

Regarding Google data, the original android records are equivalent in nature to the data used in this study (they are usually called Location History data). This information is captured through the location of the mobile phones. Actually, we have experience with these data in previous works (see, for instance, Ref. [31]), but this data is not publicly available and flows are aggregated without considering users' residential areas, hence it would be hard to develop the same analysis with such limitation. We do not discard that other treatments of this data could provide such information. In that case, the results could be obtained and reproduced equally with any of these data sources. The question is, however, the accessibility. The publicly available Google data (`https://www.google.com/covid19/mobility/`) is not so complete. It includes scores on the relative change of mobility in a specific place with respect to an unknown baseline, some information on mobility by purpose, no flows between regions or provinces, nor on the composition of the relative mobility by type.

We have updated the methods section of the paper to mention this question when the mobility data is described.

Related to the mobility, the finding seems to be that higher levels of mobility result in stronger transmission. In words of the conclusion (l.422/423): "Lockdowns slow down the progression of the disease". Isn't this intuitive?

It is intuitive, but we feel that in this way the sentence is placed out of context. The original statement was: "*With multi-seeding, the social network of each area gets hit on multiple points, creating colliding outbreaks and therefore provoking a faster spreading of the epidemic than the one produced out of a single-seed. Lockdowns slow down the progression of the disease in the different provinces and they can induce a strong relation between mobility from/to source and the peak of incidence and mortality.*" It is indeed intuitive to think that lockdowns slow the pace of spreading, however the answers to "how and how much" are less trivial. In this work, we answer to these questions by focusing on the spatial effects induced by different types of lockdown. National lockdowns freeze the spatial seeding from epidemic sources and allows to uncover the spatial seeding pattern induced by mobility. On the other hand, regional lockdowns have a totally different effect, since they may stop the importation of cases from other areas, letting the inner outbreak develop independently from the rest of the system, whereas the rest of the country keeps developing the epidemic. This leads to a totally different final picture, as shown in Fig. 4a-b, where all provinces incidence peaks are similar across the country. This is something that is observable from the empirical analysis of our work, as we show in the multivariate analysis, countries show different levels of correlation (confounding factors included), depending on the types of interventions that they implemented.

We have discussed this in the Methods section when lockdowns are described.

Some comments on the presentation and additional questions:

1. Line 59 (gridded contact): Can the authors elaborated on their idea of a gridded contact network? Is it a regular square lattice (discretization of a 2D space)? Is this a realistic assumption for a contact network in an urban area?

Yes, the gridded contact network is a regular 2D square lattice in which every node is in contact with 4 neighbors. The lattice acts as an abstraction of less regular spatial network since we are considering not a single population but full regions. Social networks can have a few long range contacts but they are eminently short range. It is probably a little more realistic to consider the networks produced with the rewiring procedure but for small rewiring probabilities. We felt that the 2D regular lattice was a good (even if strong) abstraction to test our hypothesis: introducing space in the contact networks of our sub-populations not only allows to recover the observed heterogeneity of incidence peaks when national lockdowns are issued, but they also show in the simulations an unexpected relation between the number of imported cases and the spreading speed in a single population, very differently from the widely used homogeneous mixing assumption. More realistic cases are then recovered by applying the rewiring process on the contact network and observing the same effect to vanish more and more as the rewiring probability is increased until reaching a homogeneous mixing population. We added a clarification of the "gridded" term in the same line and a reference to the "GRID" when we first introduce it three lines before.

2. The scaling of 1:500 (l.66) is not in line with the number of agents representing Madrid (12k) and Barcelona (11k).

True, these are only approximations. The values are in scale 1:500 with respect to the national statistical office, INE, population projections for Spanish provinces, hence in the model the exact number of agents in the province of Madrid is 12873 and 11047 in the province of Barcelona. We corrected these values in the text.

3. Line 82/83, 366 Remove URL from the main text

We removed the urls from this line.

4. The text of lines 95 to 99 would be more accessible as a table.

We replaced the text with Table 1 in the same place.

5. Line .108: If the Laplacian noise is applied to randomize the exact location, the scaling parameter should have units, e.g., 5km.

The Laplacian noise is added over the flows, here units are trips.

6. The statements in lines 104/105 and lines 119/120 are redundant.

We removed these lines.

7. Some sentences read strange/colloquial/too complicated or are not needed for the flow of the text. See, for instance, i. lines 133/134: "The most straightforward one is the standard in this metapopulation modeling framework and it is the so called well-mixed (WM) population". ii. line 140: "and, therefore, one may wonder what occurs when the space enters into the equation." iii. line 218: "The effect as can be seen is minimal." iv. line 235: "the mobility with the initial source stops being so important" I suggest a thorough proof-reading of the text to check the wording.

We provided more formal and easy to read explanations of these concepts in the respective lines and in other parts of the text.

8. The abbreviation WM (well-mixed) is introduced in line 134 and should be used consistently for the remainder of the text.

We replaced all the "well-mixed" terms after these lines with the respective abbreviation.

9. Fig.1: i. panel (c) The idea of a small-world network (rewired grid) is interesting. As more shortcuts are introduced, the more the peak incidences resemble the WM case (up to 50 seeds). It should be discussed why the REW leads to higher peaks compared to the WM model for 100 seeds. Is this because the existence of hubs facilitates the spread? ii. Caption: The statement The peaks and the sizes of the box plots are collected from the single simulation realizations and do not necessarily match those of the averaged curves. can be safely removed. It is clear that box plots show the statistics of an ensemble of simulations.

i. The difference of incidence peaks between the REW and the WM case here is due to the different β rates used for the two topologies. In order to keep the final size and the dynamics in similar ranges, we needed to use different β values in the WM and the GRID topologies. The REW topology simulations are run using the same parameters as the GRID topology, because the comparison is established with the pure GRID. Even so, when using more similar β rates to those of WM, as in Supplementary Figures S17, the incidence peaks with 100 seeds that we observe with the WM contact network are almost completely recovered with the REW topology. We have added a short comment on this issue in the caption of Fig. 1.

ii. Sentence removed.

10. Lines 169/170: What is the effect of the exchange of agents? Could there be an echo effect, that is, movement of infected to the location of origin of agent i? To keep the population constant, the authors could also assume a commuter model, where every agent returns to their home location.

According to mobility surveys (as the EMQ in Barcelona and the equivalent ones in Madrid or London) commuting represents between 50 and 60 % of the mobility in urban areas (see Refs [52,53]). However, the scale that we consider here is much higher since we are working with full regions. In this case, the commuting contribution is likely to be weaker and to happen mostly on the boundaries between adjacent regions. Furthermore, as we showed in a previous study (Ref [27]), weekend mobility plays a non-negligible role on the national development of the epidemics. Our model is informed with real trips observed in the two weeks of epidemic in Spain right before lockdown. Hence the flows that we observe are composed by all kinds of mobility, not only commuting. Assuming that all users, including those who moved for personal reasons, return to their origin place after 8 hours could introduce unnatural patterns of infections in our model and we feel that this would only decrease the interpretability of the model results.

Still, the echo effect, not with the same person, but interchanging infected agents between subpopulations can also appear. An infected individual traveling from i to j can produce a local outbreak in j and some of the infected in j may eventually travel to i. It occurs in a much longer time scale, in short times it is an unlike phenomenon.

11. Fig.2g: 1. Why does the WM curves do not reach a level of 1 as in the GRID cases, but saturate at a lower level? The same question applies to Fig.1d.
2. Can the curves be collapsed into one by rescaling, e.g., by the number of seeds or number of contacts? This question also applies to Figs.1a-b. This might give a handle on the mechanism and predictable impact of containment policies.

1. The difference of final size between the GRID and the WM case here is due to the different β rates used for the two topologies in the main text. The β rates used here are indeed the same used for Fig.1. In the GRID case the β is high enough to infect almost the entire population, whereas the β provided to the WM scenario is sensibly lower, hence here the epidemic dies out before reaching a comparable epidemic size as the one observed on the GRID topology. This is done in order to allow a slower contagion in the WM scenario, which is not hindered by bottlenecks as in the GRID topology. The WM model is very sensitive to the variation of the infectivity rates β, here the choice of the infectivity parameters is justified by the need for comparing the two epidemic sizes at a same finite time. If we used a higher infectivity parameter in the WM here we would not be able to clearly show this comparison made at the same time since the beginning of the simulation for the two scenarios. We have mentioned this issue in the caption of Fig. 1.

2. The number of contacts is the same in WM, REW and GRID, it is always four, so this is not a factor for the collapse of our curves even though it could play a role in a more generic setting. Still, the reviewer is right, in a single population it is possible to collapse the curves by taking into account the characteristic scale between randomly placed seeds and how it scales with the network dimension. Since this is an important question that illustrates the nonlinear relations that emerge between seeds and epidemic indicators, we have added a new Fig. 2 that shows and quantifies this effect with a paragraph explaining how to reach the collapses. The scaling arguments are explained after the first one-population results. We must thank the reviewer for this suggestion.

12. Lines 188/189: Lockdown measures might slow down the spreading, but they only stop the process if the number of infected reaches zero. With a finite residue, there will be an upsurge the moment they are relaxed or lifted.

True. We added a clarification in the line before. Here we refer only to the model abstraction and not to reality. In our model lockdowns are never lifted until the total suppression of the disease. In a more complex model we could indeed lift the lockdown and study what happens in case of disease resurgence, but this would be a study focused of interventions lifting rather then on the effect of multi-seeding and spatial dynamics.

13. Lines 212/213 (and line 180): I do not understand the notion "allowed to travel" and "allowed to enter". Either the process of mobility is stochastic or there is a fixed number of seeds. The authors should clarify how the mobility works.

The process of mobility in the model is indeed stochastic and informed by empirical mobility in the pre-lockdown period. However, in the two populations model as well as in the fixed-seeding policy, we make an iterative check on traveling agents. At every time-step agents located at population i have a probability to travel to j that is informed by empirical trips collected from the data. For every agent traveling from i to j the model makes a check on their status: if the traveling agent is infected, the number of imported cases of population j is updated, until reaching the allowed threshold of seeds per capita. Note that once the threshold is reached, mobility stops and no further agents are allowed to travel, not even those belonging to the latter set nor the susceptible ones. So the mobility in the model is stochastic, however this does not forbid us to control the entering flows in a given area in case of observing an infected traveler. We provide a more detailed explanation on the mechanisms regulating mobility and the different types of lockdown in the new version of the Materials and Methods section (subsection on Lockdown and Mobility).

14. Fig.3: i. What is the reason for dividing by 56? How does this related to three seeds? ii. The authors should try to find a different display of the data points. The log-scale for the y-axis is of little use for almost horizontal alignment of points. If that was the main information of the plots, a table stating the sigma and R^2 values would be more suitable.

i. Thanks for highlighting that this point was not clear. The reason for the choice of the number 56 is that in our model the least populated area is Ceuta, which is composed by 168 agents. In order to implement the *fixed-seeding scenario*, for each unit we had to choose a threshold in terms of imported seeds per capita. We chose to set this threshold as the local population divided by 56 because in the least populated area in the model, Ceuta, this corresponds to have 3 allowed imported cases. We could also divide by a higher number, but this would mean to have less imported seeds in Ceuta, hence more simulations producing no outbreaks, diminishing the statistics of the analyses. We proceeded to clarify this point in the text, as well as in the figure caption.

ii. In this case, in the former Fig. 3, currently Fig.4, panels a-d show the flatness of the curves is an effect that must be contrasted with the trends shown in panels e and f. Having a table with σ and R_L alone would hinder the understanding of the readers of the argument line we try to follow. The figure shows that to more trips from Madrid correspond higher incidence peaks when the *lockdown* policy is implemented. When a *fixed-seeding* policy is issued instead, the incidence peaks in all regions do not differ except for slight fluctuations. We feel that a table would not encode the information given by the heterogeneity of trips per capita coming from Madrid to each geographic unit and its relative effect on the local outbreaks severity. We have revised the text when this question is explained to make sure that the message is clear.

15. Line 223: How are the assumptions of "beta by one half, and ceasing all inter-area mobility" justified? Is the former backed up by empirical evidence on social distancing? Concerning the latter, there is surely an element of non-compliance/essential travel.

The choice of these lockdown parameters is driven by the need for abstraction: we needed to simulate a strong decrease in contacts within the sub-populations in order to observe a clear effect on the incidence peaks, possibly not affected by any demographic aspect of the provinces, hence we assumed the beta (rate of infections per contact) to decrease by half, simulating a decrease of contacts by half. A consistent decrease of transmissibility during lockdowns has been observed in a variety of countries (`https://www.nature.com/articles/s41467-021-21358-2`). The same is valid for the ceasing of all inter-area mobility. Of course in a realistic scenario essential travel would still be allowed, but in this case we wanted to check the effect of completely isolated dynamics. Hence no residual mobility was allowed.

16. Line 271/292-297: The origin zone can also be found looking at the history of the primary cases in a country, that is, the start of the transmission chains (arrival from travelers from China, physical proximity to other countries with earlier outbreaks). I do not understand the presented reasoning. Once the coronavirus arrives in a country, it spreads rapidly to all areas. To trace the point of origin, the locations/nodes of underlying network could be rescaled using a network distance. Cf. Ref.[31].

Part of the information is indeed included in the epidemic curves, although to get the full picture it must be blended with the mobility data. It is important to note that importation of cases is not strictly equivalent to develop a major local outbreak. Using mathematical jargon, it is a necessary condition but not sufficient, the two questions are not equivalent. This virus is very contagious but the arrival of the first cases do not necessarily lead to a major outbreak. For example, the recent case of a cluster of cases of SARS-Cov2 Gamma (P.1) variant in a EHPAD in the department of Creuse in France `https://www.francebleu.fr/infos/sante-sciences/variants-bresilien-et-sud-africain-du-coronavirus-deux-clusters-a-auzances-et-ahun-en-creuse-1618853326`; the first case of importation in the island of Mallorca

in February 2020 https://www.ultimahora.es/noticias/local/2021/02/06/1236407/coronavirus-baleares-cumple-ano-llegada-del-virus.html; or the cluster developed few weeks before in the French Alps https://academic.oup.com/cid/article/71/15/825/5819060. In all these cases, despite few initial contagions among the residents of the EHPAD, the family of the English man in Mallorca and friends in the French Alps, the epidemics in these places developed only one month later, when many more cases started to arrive. In this sense, including mobility history into the method is key to understand if effectively a first cluster of contagions was followed by exportation of cases to other regions or if the cluster was efficiently confined, especially considering the high uncertainty on the reported cases from the first wave. Our method is able to unveil the correlations between major local outbreaks, it can be conceptually related to Granger causality but mediated by the mobility data.

It is important to mention that our approach is different from the early works by Gautreau and by Brockmann of Refs. [33,43,44], in which the main focus was set on the arrival of the first seed. Our main result is that the severity of local outbreaks (full curve) is related in a non trivial way to the number of arriving seeds, not with the arrival of the first one that may or not ignite the epidemic. In this sense, both approaches are complementary: while Gautreau et al and Brockmann et al. designed a method to predict the arrival of the disease at international scale in terms of wavefronts, we design a new method to predict the severity of the outbreak at finer geographical scales in terms of the in-seeding fraction over the local population. We explain this point better now in the abstract, intro and conclusions.

17. Lines 447-453: I would have liked to see more on the impact of regional lockdowns considered in the simulations of the current study (cf. Fig.8 for Madrid). This would be insightful for more targeted intervention strategies in the future.

Spatial heterogeneity in the lockdowns can, of course, be included into the model. Still, in the analysis we wanted to connect the local epidemic characteristics and the number of arriving seeds in the early stages. This is why we have cut completely the mobility after the lockdown starts, to prevent the further arrival of seeds and to have a clear defined variable. In a more realistic setting, it is possible that partial lockdowns could reduce the inflow of seeds but not eliminating it. This could be an interesting scenario for a subsequent work, especially if the arrival rate is low enough as to separate time scales and allow for a partial recovery of the local outbreak before new waves start. The goal here was to understand the basic effects of multiseeding as a first step towards more realistic modeling, in this sense we believe that the work is now quite complete.

To sum up, while the implementation of the methodology of the study looks sound from a technical point of view, but there are open questions on the parameter selection and network realization. More severely, I fail to see the key innovation of the investigation at the moment. However, I would invite the authors to clarify the scope, key findings, and purpose of the study in a revision. This could include, for instance, a quantitative analysis of the impact of different levels of lockdown: a reduction of X percent in mobility delays the peak by a factor of Y. At this stage, too many things are unclear or seem to be intuitive and of qualitative nature.

We hope that the new version of the manuscript explains better which is the originality of this work with respect to previous papers in the literature, how it complements previous approaches and the strength of our main results.

Reviewer #2:

In this manuscript the authors investigate the role played by human mobility in shaping the heterogeneity observed in the disease incidence across different locations, showing how mobility patterns could become especially relevant in presence of strong restrictions on individual movements and contacts. COVID-19 is used as an illustrative example for the carried out analysis.

The manuscript relies on multiple approaches. The main one consists of a modeling analysis based on a metapopulation SEIIR model, accounting for multi-seeding (multiple introduction of the infection in a population) and realistic mobility patterns derived from digital data records (GPS records from the use of mobile phones). The model is used to highlight that multi-seeding and a spatially structured contact network may explain the geographical heterogeneity observed in COVID-19 incidence and mortality in five European countries. The analysis suggests that highly connected regions are more likely to experience larger outbreaks before control interventions are implemented. Statistical analyses are also provided using real COVID-19 data retrieved from different countries to highlight the relation between the mobility patterns and the data on mortality/incidence peaks.

I personally find this article technically sound and quite interesting, providing new insights to the spread of epidemics with potential implications for public health policies. Specifically, I really enjoyed the approach adopted by authors to identify the geographic area that likely acted as the initial source of infection across

different countries. As such, I recommend the paper for publication in PLOSCB, after few but essential revisions.

My main concern regards how the methods are described in the main text.

In particular, a couple of assumptions are not sufficiently clear to me and some technical details are reported in results instead of in the methods, making it a bit hard-working to assess the appropriateness of specific analyses and hindering the overall understanding of the paper. For instance, I didn't get how and why different digital data were used for the different countries (Kido for Spain and Cuebiq for others?)

First of all, we would like to thank the reviewer for the positive and useful comments and suggestions.

The choice of the data is mainly driven by the availability of mobility records and statistical power. We started studying this effect in Spain with mobile phone records, which cover a little over 20% of the population. We did not have, however, access to equivalent datasets for the other countries. Therefore, we relied upon the app generated data of Cuebiq. The fraction of population covered is much smaller, but the effect of multiseeding is still visible. Furthermore, in the case of Spain, where we have data from the two sources, we observe that the two types of data provide consistent results (Fig.S12). We have explained this better in the Data subsection of Methods.

, why 4 contacts per individual were assumed in the simulation

We use a grid with 4 neighbors for representing a social contact network in space extremely influenced by the distance, we kept the same number in the other networks as well to prevent variations in the number of contacts. Differences in the number of contacts across networks can strongly affect the dynamics of the disease. Further studies could explore the effect of multi-seeding in less homogeneous networks, however we felt that, for the scope of this study, such simplifying assumption was justified. This point is now treated with more detail in the model definition section.

and how the timing for the lockdown was assumed/simulated. I feel that these assumptions do not strongly affect the general findings of the paper, but they should be better specified in the text.

The timing of the lockdown is based on the reaching of a threshold imposed on Madrid in terms of cumulative cases. When Madrid reaches the first 2000 cumulative cases, the lockdown is issued. This was thought initially to resemble what actually happened in Spain during the first lockdown, when Madrid reached the first 100 cases on March 6, starting the debate on a possible national lockdown, which one week later was effectively issued. The case is that in the model there is no space for undetected cases, hence we could not use a similar value to set our threshold, moreover we needed to let the disease spread long enough to provoke measurable outbreaks in all the areas. The effect of different thresholds is further explored in the last part of the work, but the general results of the model are not strongly affected. We discuss these questions now in the main text when first introducing the national lockdown policy in the Methods Section.

I also suggest the authors to provide an overview of the rationale of the entire analysis at the beginning of the methods, highlighting the different approaches and data used for this study.

Following the suggestion of both reviewers 1 and 2, we have added a more clear explanation on the concepts involved, the rationale, methods and the novelty of the study at the end of the introduction and Methods sections.

Minor comments:

I suggest to use the term "homogeneous mixing" when defining the "well mixed" network

We added the term in the first definition of the well-mixed network.

I assume that the "GRID" notation that appears in Figure 1 refers to the "lattice network" configuration mentioned in the text. Please use the same term for the text and figures. The authors also say a lattice as contact network (see below for details) but no details are available on this in the main text or in the Supplement.

We proceeded to replace "lattice" by grid in a consistent way, except in the first mention of the network, in which another reviewer asked to define it explicitly and we needed to use such term. We removed the "see below for details" since the grid is effectively a 2D regular lattice with no further particular details.

When considering a lattice structure for the network, the authors state that "every individual is a node and can interact only with her nearest neighbors". Do you mean that, under this assumption, individuals have only contacts with those living in adjacent cells on a grid representing different provinces? Please clarify the point.

The network represents the contacts within provinces, which are relatively extensive. The grid topology gives importance to the distance, the rewired networks dilute this effect and the WM directly ignores space. The grid topology thus can be seen as cells dividing the space where people live, clearly it is an exaggeration of the relevance of space in social networks within the province but it is a limit case. We find more intuitive to think of the grid as nodes corresponding to individuals instead of cells, but one can easily imagine the same topology with agents living in cells instead. Note that the provinces and connections between them are always based on mobility data and they are not changed, except when lockdown is applied. We have modified the description of the social networks to explain better this point.

A better description of what sigma represents (standard deviation?) and how it could be interpreted would be helpful (e.g. when sigma is low, no marked differences could be appreciated between different geographical locations)

We thank the reviewer for highlighting this lack of clarity. Yes, sigma is the standard deviation of incidence peaks. We added some clarifications in the caption of Figure 3 and in its first definition in the text.

Fig 1: - In panels c) and d) the order of the legend is a bit confusing. For the three rewired network models (REW), the probabilities assigned are not in increasing nor decreasing order. Would be better to put in order the three REW (p=2x10**-2, p = 10**-2 and p = 10**-3). - Panel d) is hardly visible.

We have updated the Figures.

Caption of Fig 2: "a function of time step" should read "a function of time"

We corrected this sentence.

Caption of Fig 3: - "The threshold is the destination population divided by 56 so that at least three seeds can travel." Why this threshold? Where is the relation between the 56 factor and the 3 seeds? Explain better.

Thanks for letting us know that this aspect was not clear. In our model, the least populated area is Ceuta. The number of agents is obtained by dividing the population by that 56 factor. The reason for such particular number is that in order to implement the *fixed-seeding scenario* for each unit, we choose a threshold in terms of imported seeds per capita. We chose to set 56 because in Ceuta this corresponds to have 3 allowed imported cases. We proceeded to clarify this point in the text, as well as in the figure caption.

- "Finally, in panels e and f, the simulations are repeated with a national lockdown applied when Madrid arrives at 2000 cases." I guess the 2000 cases are cumulative. Please clarify.

Yes, 2000 refers to cumulative number of cases. We have clarified this in the same line.

Finally, please note that Figures S4-S11 from the supplementary material have all the panels with different ranges for both x and y axis, which hinders the comparison between them.

Figures S4-S11 are not showed for comparison purposes, which would not be feasible at a glance, but only to show how the epidemic data is treated: how incidence peaks and onsets are set along all the curves in every unit area. Using a same scale for all areas would hinder the observation of the trend in most of the least hit geographic units.

Reviewer #3:

The manuscript provides an analysis of the spatial spread of the COVID-19 epidemic in 5 countries in Europe during spring 2020. Focus of the analysis is the role of multi-seeding coupled with social distancing on the spatial heterogeneities in incidence and mortality. The study leverages on high resolution mobility data collected by means of mobile devices. The analysis of the COVID-19 epidemic is complemented by the analysis of simplified synthetic spatial systems to better highlight the role of the different ingredients (multi-seeding, local network of contacts, timing of the intervention).

The study tackles an important fundamental question, and the case study of the COVID-19 epidemic is highly relevant. The analysis presented provides important insights on the early-stage spatial propagation of COVID-19 in the European countries considered. On this basis, I feel that the manuscript has the potential to provide a nice contribution to PLOS Computational Biology. At the same time, however, the writing and the presentation of the work is unclear in many parts. With this respect, I believe that there are some major points that would need to be addressed.

The manuscript includes a Methods section, that is presented before the Results section. This part is too brief and overall unclear. In fact, it does not present all methods. Part of the analysis is presented throughout the Results section. As a result, the overall plan of the work is not clear, as well as its

objectives. The study is articulated in several analyses, including mechanistic simulations of synthetic population systems with increasing level of complexity and the study of the covid-19 epidemic with data analysis techniques. The needs for combining all the different steps should be better explained since the beginning.

First of all, we would like to thank the reviewer for the comments and suggestions that will contribute to improve the manuscript.

In this point, the reviewer is in agreement with the previous reports. We have updated the introduction and the methods section to explain more clearly the objectives of the work, how these are not covered in the literature and how we attain them. In the particular case of the methods section, we provide now more details on the models including the contact networks, the implementation of mobility and of lockdowns. In a second step, we have also updated the description of the data (mobility and epidemics), and of the data treatment, closing with the metrics used to detect what we called before epidemic region of origin, now epidemic epicenter.

Later in the Results section, authors present the use of LOESS to fit the relationship between peak incidence and mobility. This analysis should be better presented, and the choice of the LOESS method should be better motivated.

The relation between incoming mobility per capita from the epidemic epicenter and the local incidence peaks is not necessarily linear. In this sense, we preferred to use the LOESS method to fit this trend in order not to assume any particular functional relation between couples of variables. As can be seen from the results in Fig.4-5, this relation in the data is not trivial and it is sensibly affected by many possible confounding factors. This choice and the non parametric nature of LOESS is now better explained in the methods section of manuscript, when this method is firstly introduced.

In section Effects of multi-seeding in the first pandemic wave author present a correlation analysis between mobility flows and peak incidence. They write We start our analyses by showing the connection between mobility from the region or province of origin to every destination and epidemic features such as the maximum local incidence or mortality in the area of destination. The region of origin authors refers to is defined as the one that maximize the correlation. However, if I correctly interpreted this part, no validation is provided on the fact that the specific region is the actual origin of the countrys epidemic. To avoid any ambiguity, authors should use a different notation. At the same time, they could discuss their findings in light of available information on key epidemic events or first epidemic clusters detected in the countries. This is done with some extent, but it should be expanded and relevant citations should be provided. For instance, the first cluster identified in Germany was in Bavaria (`https://www.thelancet.com/journals/laninf/article/PIIS1473-3099(20)30314-5/fulltext`). Likely, similar information is available for other countries.

We have now added in the text a discussion on the historical evolution of the pandemic in the five countries and how it correlates with our findings on the potential epicenter. This helps to improve the argument line of the manuscript. Still, it is important to note that, even though they can be similar, the epicenters are not necessarily corresponding to the first regions detecting cases. The initial cases may recover without expanding the disease further away. These epicenter regions are characterized by a combination of early local outbreaks and good connections to the rest of the country, which makes them most likely responsible for the full country spreading. We have clarified this point in the results section and conclusions.

We proceeded to replace the word "origin" with "epidemic epicenter" to prevent confusions between the areas with the first cases of the epidemic in the country and those that maximize the R^* score in our method.

What is the role played by the population size of a destination region? This should impact the number of new cases at the peak, but also the timing of the peak. Would that be possible to provide a mechanistic understanding on its role and its interplay with mobility?

Yes, indeed this is what we did in Figure S3a-b. We took Madrid and Valencia as test populations and instantiated in them the same absolute number of seeds at $t = 0$. Madrid has a larger population, hence the same number of seeds provokes a less severe outbreak in terms incidence peaks with respect to what happens in Valencia, see Fig.S3a. In Fig.S3b, we rescale the number of seeds with the ratio between the populations to produce in Valencia curves with the same outbreak severity that we observed in Madrid in Fig.S3a. This effect makes population size on the destination region quite important in multiseeding analysis. This is why we always weight the imported mobility from the epidemic epicenter on the local population. The effect of every arriving seed is much larger in small populations like Soria than in large metropolises as Barcelona or Madrid. Mechanistically, this can be explained by a thought experiment based on the superior panels of Fig. 3, where we can see the effect that extra seeds have in a grid. If

we considered a grid twice as big but with the same number of seeds, the impacts of the seeds in terms of the times to the peak and the height of the peaks would be smaller and similar to divide the number of seeds in one half. If fact, as explained in the discussion on the scaling in one population, the time to the peak in a grid goes as $t_{\text{peak}} \sim (N/s)^{1/2}$ and the height of the peak $I_{\text{max}} \sim (N/s)^{-1/2}$, where N is the total population and s the number of seeds. The important variable in this case is the ratio between N and s that determines the characteristic distance distance between randomly located seeds in the grid, $\sim (N/s)^{1/2}$. We have mentioned this question now in the text when the scaling is discussed.

Figure 1: It seems to me that results with lower beta are quite interesting and would deserve to be presented in the main paper.

The results of the model with lower beta confirm the findings of the main manuscript. We agree with the reviewer that they are quite interesting. Still adding them in the different figures would make them quite dense and harder to follow, this is why we decided to include them in the SI. If possible, unless an alternative is suggested we would prefer to maintain this choice.

The sentence Therefore, mobility yields a first-order effect on the local incidence peaks due to the multi-seeding, as we observed empirically, and synchronized and homogeneous lockdowns allow to observe this effect, potentially saving many lives. is unclear.

We have modified it.

The area considered in the analysis are either province or regions according to the country. However, authors use sometimes the term "province" to generically refer these areas (for all countries) thus creating confusion (e.g. caption of Figure 7)

Thanks for noticing the potential confusion. We use now region or (unit) areas when possible instead of provinces. Province remains only for the areas of Italy and Spain.
